# Hetero-trans-β-Glucanase Produces Cellulose–Xyloglucan Covalent Bonds in the Cell Walls of Structural Plant Tissues and Is Stimulated by Expansin

**DOI:** 10.1016/j.molp.2020.04.011

**Published:** 2020-07-06

**Authors:** Klaus Herburger, Lenka Franková, Martina Pičmanová, Jia Wooi Loh, Marcos Valenzuela-Ortega, Frank Meulewaeter, Andrew D. Hudson, Christopher E. French, Stephen C. Fry

**Affiliations:** 1 The Edinburgh Cell Wall Group, Institute of Molecular Plant Sciences, School of Biological Sciences, The University of Edinburgh, Edinburgh EH9 3BF, United Kingdom; 2 Institute of Quantitative Biology, Biochemistry and Biotechnology, School of Biological Sciences, The University of Edinburgh, Edinburgh EH9 3BF, United Kingdom; 3 BASF, BBCC Innovation Center Gent – Trait Research, 9052 Gent (Zwijnaarde), Belgium; 4 Zhejiang University-University of Edinburgh Joint Research Centre for Engineering Biology, Zhejiang University, Haining, Zhejiang 314400, China

**Keywords:** cell wall, cellulose, hemicelluloses, hetero-trans-β-glucanase, hetero-transglycosylation, xyloglucan

## Abstract

Current cell-wall models assume no covalent bonding between cellulose and hemicelluloses such as xyloglucan or mixed-linkage β-d-glucan (MLG). However, *Equisetum* hetero-trans-β-glucanase (HTG) grafts cellulose onto xyloglucan oligosaccharides (XGOs) – and, we now show, xyloglucan polysaccharide – *in vitro*, thus exhibiting CXE (cellulose:xyloglucan endotransglucosylase) activity. In addition, HTG also catalyzes MLG-to-XGO bonding (MXE activity). In this study, we explored the CXE action of HTG in native plant cell walls and tested whether expansin exposes cellulose to HTG by disrupting hydrogen bonds. To quantify and visualize CXE and MXE action, we assayed the sequential release of HTG products from cell walls pre-labeled with substrate mimics. We demonstrated covalent cellulose–xyloglucan bonding in plant cell walls and showed that CXE and MXE action was up to 15% and 60% of total transglucanase action, respectively, and peaked in aging, strengthening tissues: CXE in xylem and cells bordering intercellular canals and MXE in sclerenchyma. Recombinant bacterial expansin (EXLX1) strongly augmented CXE activity *in vitro*. CXE and MXE action in living *Equisetum* structural tissues potentially strengthens stems, while expansin might augment the HTG-catalyzed CXE reaction, thereby allowing efficient CXE action *in muro*. Our methods will enable surveys for comparable reactions throughout the plant kingdom. Furthermore, engineering similar hetero-polymer formation into angiosperm crop plants may improve certain agronomic traits such as lodging tolerance.

## Teaser

Studying a hetero-transglucanase showed that cellulose and mixed-linkage glucan can be covalently linked to the hemicellulose xyloglucan in native plant cell walls. These reactions increase with tissue age and predominate in structural tissues and in cells surrounding cavities, suggesting a role in tissue strengthening. Expansin strongly augments cellulose hetero-transglucosylation but has no effect on mixed-linkage glucan.

## Introduction

Plant cells are surrounded by cell walls, which are composites of complex polysaccharides and crucial for plant function and survival (Popper et al., 2011). Current structural models of plant primary cell walls state that cellulose microfibrils form a load-bearing network, while hemicelluloses tether or fold into this structure (Fry, 1989, Park and Cosgrove, 2012) and strongly influence the properties of the cellulose fraction (Li et al., 2015). The hemicellulose–cellulose network is embedded in the rest of the matrix of pectin and hemicelluloses (Carpita and Gibeaut, 1993, Cosgrove, 2018). Furthermore, plant cell walls contain proteins, such as expansins, which act on the polysaccharide fraction and transiently disrupt hydrogen bonds between hemicellulose and/or cellulose molecules (Cosgrove, 2015). In contrast, current cell-wall models assume no covalent links between the cellulose and hemicelluloses. However, recent *in-vitro* studies have revealed that certain hetero-transglucanase activities exist that can catalyze the cleavage of a cellulose molecule (donor substrate) followed by its covalent attachment to a xyloglucan-oligosaccharide (XGO) (acceptor substrate; Simmons et al., 2015, Shinohara et al., 2017). This hetero-transglucosylation differs from homo-transglucosylation of hemicelluloses (Fry et al., 1992), where the donor and acceptor substrate, usually xyloglucan, are chemically identical (Franková and Fry, 2013).

Homo-transglucosylation of cell-wall hemicelluloses is widely studied because of its relevance for plant function and because land plant genomes typically encode ∼30 xyloglucan-acting trans-β-glucanases (XTHs) (Yokoyama et al., 2010). The xyloglucan/xyloglucan endotransglucosylase (XET) reaction catalyzed by such enzymes participates in cell-wall formation and loosening (Thompson and Fry, 2001, Van Sandt et al., 2007), vascular tissue development (Matsui et al., 2005), fruit growth and ripening (Han et al., 2015), gravitropic responses (Nishikubo et al., 2007), and sensing and counteracting metal stress (Zhu et al., 2012).

**Figure 1:**
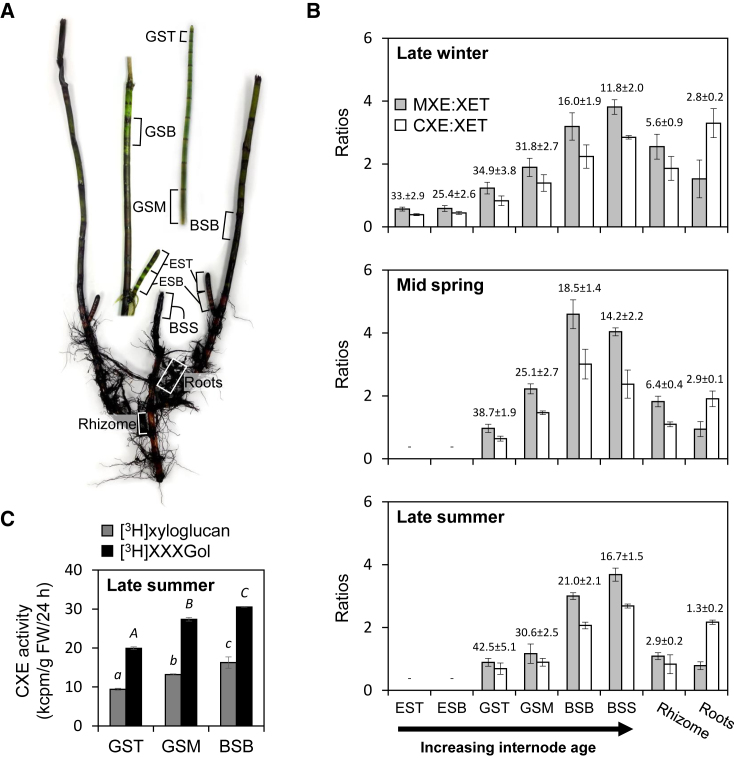
Extractable Transglucanase Activities from Various *Equisetum* Parts. **(A)** Representative *Equisetum fluviatile* plant, where parts of different age used for *in-vitro*, *in-situ*, and *in-vivo* studies are marked: tip and base of emerging young shoot (EST, ESB); tip, middle, and base of green shoot (GST, GSM, GSB); blackish shoot base above water level (BSB); root-free blackish submerged shoot base (BSS). Submerged rhizome and outgrowing roots are marked. Scale bar, 5 cm. **(B)** XET, MXE, or CXE reaction products generated *in vitro* by organ extracts were quantified after 24 h of incubation (linear reaction range). XET and MXE activity were assayed with soluble donor substrates (xyloglucan, MLG), while filter-paper was the donor for measuring CXE activity. The histograms show MXE and CXE activity relative to XET activity (MXE:XET and CXE:XET ratio) in extracts from shoot parts of different age, rhizomes, and roots collected during different seasons. Absolute XET values are shown above columns in kcpm/g fresh weight/24 h ± SD (where kcpm = 10^3^ counts per minute). Acceptor substrate: [^3^H]XXXGol. “-” indicates that the plant part did not occur during this season. **(C)** CXE activity in extracts of different shoot parts collected in late summer and using [^3^H]XXXGol or [^3^H]xyloglucan as acceptor substrates. Statistically significant differences (*p* < 0.05) among different extracts are indicated by lowercase ([^3^H]xyloglucan as acceptor) or uppercase italic letters ([^3^H]XXXGol as acceptor). *n* = 3 ± SD.

In contrast, hetero-trans-β-glucanase (HTG), an acidic GH16 (family-16 glycosylhydrolase) enzyme found in the evolutionarily isolated genus *Equisetum* (horsetails; Figure 1A), preferentially grafts mixed-linkage β-d-glucan (MLG) or cellulose, but also xyloglucan, onto an XGO acceptor, thus exhibiting MXE (MLG:xyloglucan endotransglucosylase), CXE (cellulose:xyloglucan endotransglucosylase), and XET activities, respectively (Simmons et al., 2015, Simmons and Fry, 2017). *Equisetum* does possess the three relevant polysaccharides: cellulose, xyloglucan, and MLG (Fry et al., 2008a, Sørensen et al., 2008). Another GH16 protein acting on cellulose is *At*XTH3 from *Arabidopsis thaliana*. Besides its predominant XET activity, *At*XTH3 covalently links amorphous cellulose to either XGOs or cello-oligosaccharides (Shinohara et al., 2017). Barley *Hv*XET5 was shown to exhibit appreciable transglycanase activity with soluble cellulose derivatives as donor substrate: for example, with hydroxyethylcellulose the rate was ∼44% of XET activity; cellulose itself was not tested (Hrmova et al., 2007). Similarly, an XTH from germinating nasturtium (*Tropaeolum majus*) seeds (*Tm*XET(6.3)) grafts xyloglucan or hydroxyethylcellulose onto XGOs and cello-oligosaccharides (Stratilová et al., 2010, Stratilová et al., 2019).

This makes *Equisetum* HTG the only enzyme known that (1) grafts insoluble cellulose onto XGOs and (2) prefers cellulose over xyloglucan as donor substrate *in vitro*, suggesting that cellulose–xyloglucan hetero-polymer formation occurs *in vivo*. HTG was shown to catalyze the formation of MLG–XGO hetero-products *in vivo* (Mohler et al., 2013), and the MXE:XET activity ratio increased with increasing tissue age (Fry et al., 2008b), suggesting that HTG plays a strengthening role in aging *Equisetum* tissues. However, it is unknown (1) whether cellulose–xyloglucan bonds are formed in native plant cell walls, (2) in which tissues hetero-polymer formation (cellulose–XGO or MLG–XGO products) is localized, and (3) what its functional roles could be. To address this, we developed a set of methods allowing us to assay different (hetero-)transglucanase actions simultaneously in freshly cut plant tissues. This overcomes the limitations of *in-vitro* studies assaying a cell-wall enzyme's “activity” (separated from its natural substrates and the cell-wall environment). Measuring the “action” of an enzyme (when still within the cell wall and utilizing native donor substrates) is crucial to evaluating its physiological role and thus relevance for cell-wall metabolism and plant morphology. Our experiments take advantage of the well-documented observation that transglucanases can be studied in experiments in which labeled acceptor oligosaccharides are supplied (Smith and Fry, 1991, Mohler et al., 2013, Rydahl et al., 2018).

*In vitro*, HTG acts more readily on cellulose after this donor substrate has been rendered accessible by alkali treatment. This suggests that native cellulose may be relatively unavailable to the enzyme *in vivo* unless the hydrogen bonds conferring the crystalline structure of the microfibrils have been disturbed. Expansin is a natural agent that might achieve this disruption of cellulose (Cosgrove, 2015) *in vivo*. Therefore, we also tested whether expansin can enhance CXE product formation in *in-vitro* assays.

Using *Equisetum* and HTG with its relatively high CXE activity as a model system to study hetero-transglucosylation is advantageous because *Equisetum* stems elongate relatively quickly and comprise numerous internodes, which represent a segmented gradient of increasing tissue age from tip to bottom. This allows us to study all tissues at various developmental stages within a single plant.

Here we report CXE action *in situ*, providing evidence that cellulose undergoes non-hydrolytic, enzyme-catalyzed covalent modifications in native plant cell walls. We demonstrated that CXE action occurs around cavities and that it peaks in mature *Equisetum* shoots, while MXE action is restricted to strengthening tissues. Furthermore, we revealed that expansin strongly increases CXE activity at an apoplastic pH.

## Results

### Evidence for CXE Action in Native Plant Cell Walls

Monitoring extractable transglucanase activities from various *Equisetum* tissues by *in-vitro* assays using soluble xyloglucan or MLG or insoluble cellulose as donor substrates and [^3^H]XXXGol as acceptor showed that both the MXE:XET and the CXE:XET ratio increased with shoot age independently of the season. Ratios peaked in blackish internodes (Figure 1B). This positive correlation between relative transglucanase activities and shoot maturity is caused by (1) decreasing XET activity of standard XTHs and, possibly, HTG and (2) increasing MXE and CXE activity of HTG with age. In both rhizomes and roots, extractable MXE and CXE activity exceeded XET throughout the year. MXE generally exceeded CXE in shoots and rhizomes, but CXE dominated in roots (Figure 1B); however, absolute extractable transglucanase activities from roots and rhizomes were much lower than from shoots (Figure 1B).

We further showed that *Equisetum* extracts exhibit high CXE activity *in vitro* with [^3^H]xyloglucan polysaccharide as acceptor substrate (∼50% of the rate with [^3^H]XXXGol) and activity increases significantly with tissue age (Figure 1C). Polymeric xyloglucan is presumably the natural substrate *in vivo*. Nevertheless, our results confirm that XGOs are still suitable for measuring (hetero-)transglucosylation. XGOs are advantageous because they do not strongly hydrogen-bond to the cell-wall matrix and thus produce fewer artifacts.

**Figure 2:**
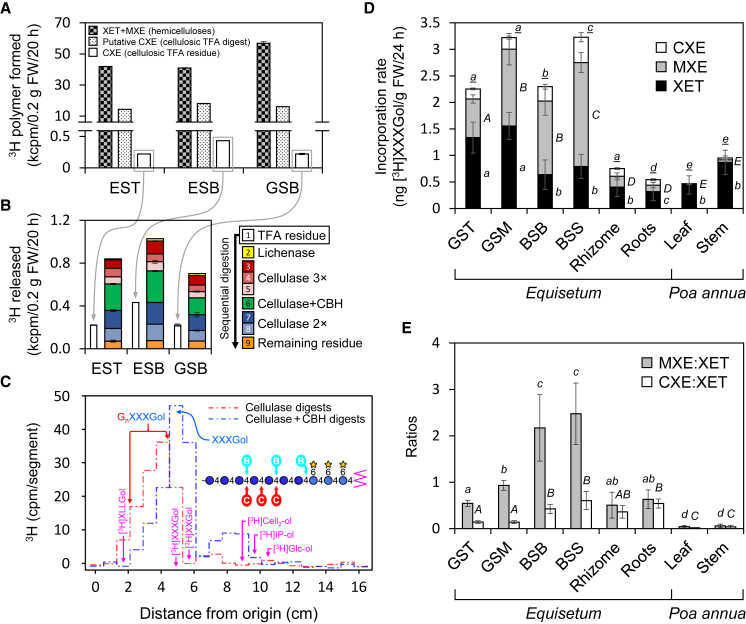
Transglucanase Action Products Formed *In Vivo* in *E. fluviatile* Tissues. **(A)** January tissue slices were fed with [^3^H]XXXGol for 20 h, and its covalent incorporation into 6 M NaOH extractable hemicelluloses (xyloglucan or MLG; XET + MXE product) or alkali-inextractable cellulose (putative CXE product) was quantified (linear reaction range). Putative CXE products were then treated with TFA (2 M, 120°C, 1 h) and remaining insoluble ^3^H (TFA residue) was reassayed (“deeply sequestered” cellulose–[^3^H]XXXGol CXE products). **(B)** Sequential digestion of the TFA-resistant CXE product by lichenase (3 h), cellulase (3 × 3 h), cellulase + cellobiohydrolase (3 h), and 2× cellulase (24 and 48 h). **(C)** Thin-layer chromatography fingerprints of the digestion products formed from the TFA residues of *in*-*vivo*-generated cellulose–[^3^H]XXXGol by *in-vitro* digestion with xyloglucan-inactive cellulase (EST, ESB, and GSB; pooled digests 3–5 from **(B)**] and the subsequent synergic action of xyloglucan-inactive cellulase + cellobiohydrolase (EST, ESB, and GSB; digest 6). G_*n*_XXXGol, hybrid cello-oligosaccharide–xyloglucan heptasaccharide conjugate (where G_*n*_ = number of glucose units in the cellulosic tail; *n* = 1–4). The structural diagram shows the expected sites of attack by “C” xyloglucan-inactive cellulose and, subsequently, “B” cellobiohydrolase. Purple arrows show the positions of markers; non-standard abbreviations: IP-ol, isoprimeveritol; Glc-ol, glucitol. **(D and E)** Similar experiment as in **(A)**; however, MXE and XET action were distinguished by lichenase digestion of alkali-extracted hemicelluloses and expressed as amount of [^3^H]XXXGol incorporated into the HTG products. XET, MXE, and CXE action were thus separately quantified in *Equisetum* tissues and *P. annua* (grass) leaves and stems (*n* = 4, ±SD). In **(D)**, statistically significant differences (*p* < 0.05) between XET, MXE, or CXE in different plant parts are indicated by lowercase, uppercase, or underlined italic letters, respectively; in **(E)**, significant differences (*p* < 0.05) between MXE:XET and CXE:XET ratios in different plant parts are indicated by lowercase or uppercase italic letters, respectively.

We then developed an assay to demonstrate transglucanase action of HTG in cell walls from freshly cut tissues, allowing us to quantify hetero-polymer formation between endogenous cell-wall cellulose and exogenous radiolabeled XGOs (Figure 2). In the same living plant parts, XET and MXE action were also quantified (by the method of Mohler et al., 2013) (Figure 2A, “hemicelluloses”). Radioactivity left in the cellulosic cell-wall fraction after thorough hemicellulose removal with NaOH and lichenase—each of which on its own efficiently removes MLG and thus MXE products from cell walls (Figure 2D and Supplemental Figure 1)—was interpreted as putative cellulose–[^3^H]XXXGol conjugates, i.e., products of CXE action. Hot trifluoroacetic acid (TFA) released 97.5%–98.5% of the ^3^H from these putative CXE products (Figure 2A), as expected, since TFA can hydrolyze the [^3^H]XXXGol moiety from the outermost peripheral [^3^H]XXXGol–cellulose conjugates as they are fully exposed to hot TFA. Evidence of CXE action in the plant tissues was obtained by detection of a proportion of TFA-resistant cellulose–[^3^H]XXXGol, evidently sequestered within microfibrils (Figure 2A). The hot TFA would have removed any remaining hemicelluloses (MXE and XET products). The observation of TFA-resistant ^3^H furthermore suggests that HTG can attach [^3^H]XXXGol covalently not only to peripheral cellulose microfibrils but also to cellulose chains within the microfibrils. The latter action yields TFA-resistant [^3^H]XXXGol–cellulose conjugates. Control groups showed only very low values when assayed for ^3^H (<20 cpm).

**Figure 3:**
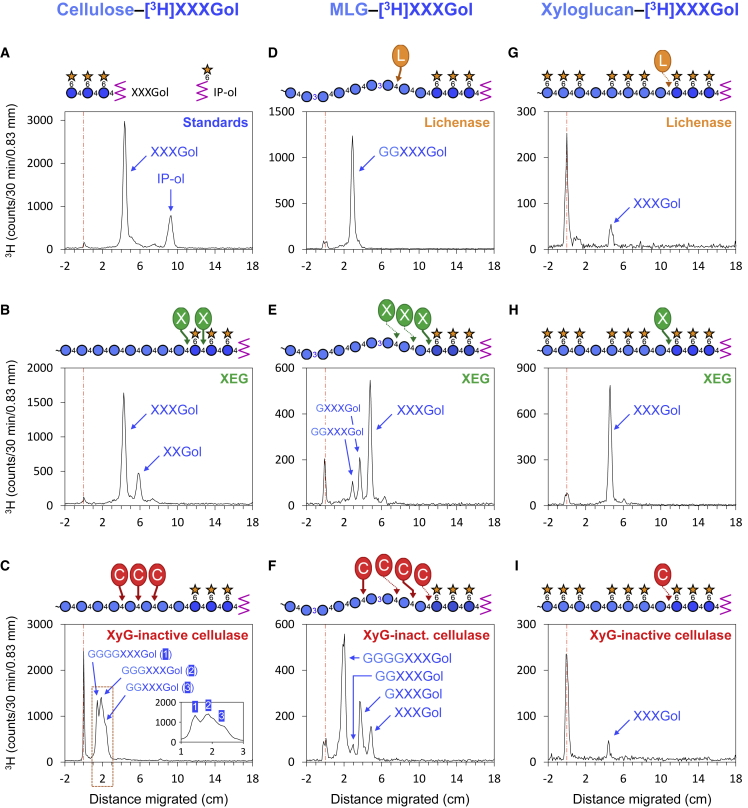
Thin-Layer Chromatographic Profiling Diagnostic Fingerprints of *In Vitro-*Formed XET, MXE, and CXE Products. Polymer–[^3^H]XXXGol conjugates, prepared enzymically *in vitro*, were digested with commercial enzymes and the products analyzed by thin-layer chromatography. Circled letters C, L, and X with heavy arrows indicate the expected sites of attack by xyloglucan-inactive cellulase, lichenase, and xyloglucan endoglucanase, respectively. Circled letters with faint arrows indicate unexpected sites of (slight) attack, possibly due to contaminating enzymes. **(A)** Markers were [^3^H]XXXGol and [^3^H]isoprimeveritol (IP-ol). Other profiles show the products formed from: the CXE product, cellulose–[^3^H]XXXGol **(B and C)**; the MXE product, MLG–[^3^H]XXXGol **(D–F)**; and the XET product, xyloglucan–[^3^H]XXXGol **(G–I)**—by lichenase **(D and G)**, xyloglucan endoglucanase **(B, E, and H)**, or xyloglucan-inactive cellulase **(C, F, and I)**. On the structural diagrams, circle denotes glucose residue, star denotes xylose residue, and zigzag denotes [^3^H]glucitol.

Xyloglucan-inactive cellulase (EC 3.2.1.4), catalyzing the endo-hydrolysis of (1,4)-β-d-glucosidic linkages in cellulose, removed little of the TFA-resistant radioactivity, but more of it was solubilized by the synergic action of cellobiohydrolase (releasing cellobiose from cellulose and cello-oligosaccharides as small as cellotetraose) and xyloglucan-inactive cellulase (Figure 2B). The products solubilized by these enzymes corresponded on thin-layer chromatography to GGXXXGol, GGGXXXGol, and GGGGXXXGol (Figure 2C), structures obtained after cellulase treatment of *in-vitro*--formed authentic CXE products (Figure 3). The results confirm that HTG covalently targets *Equisetum* cellulose in native plant cell walls.

### CXE Action Increases with Tissue Age

To expand the above experiment (Figure 2A–2C), we assayed further *Equisetum* tissues and estimated the amount of [^3^H]XXXGol incorporated into the tissue. We also tested tissues of the grass *P. annua* (Figure 2D), which exhibits all the HTG substrates (xyloglucan, MLG, cellulose) but is not known to possess HTG. Furthermore, we quantified XET and MXE action individually, allowing us to determine MXE:XET and CXE:XET action ratios (Figure 2E). Again, CXE action was measurable in all *Equisetum* plant parts tested, albeit lower than XET or MXE. Highest CXE action was measured in blackish shoots collected from above or below the water level (Figure 2D), giving CXE:XET ratios of 0.4–0.6 (Figure 2E). Both the amount of CXE action (Figure 2D) and CXE:XET ratios (Figure 2E) increased significantly with increasing shoot age. Lowest CXE values occurred in roots, where, however, the CXE:XET ratio was higher than in young shoots owing to low XET action in roots. CXE action correlated with MXE action and the latter was lowest in the tip of young green shoots and highest in old blackish shoot parts (Figure 2D), giving MXE:XET ratios of ∼0.5 and ∼2.4, respectively (Figure 2E). *P. annua* stems and leaves only exhibited appreciable XET action, not hetero-transglucosylation (Figure 2D).

### MXE and CXE Action Are Highly Localized in *Equisetum* Shoots

**Figure 4:**
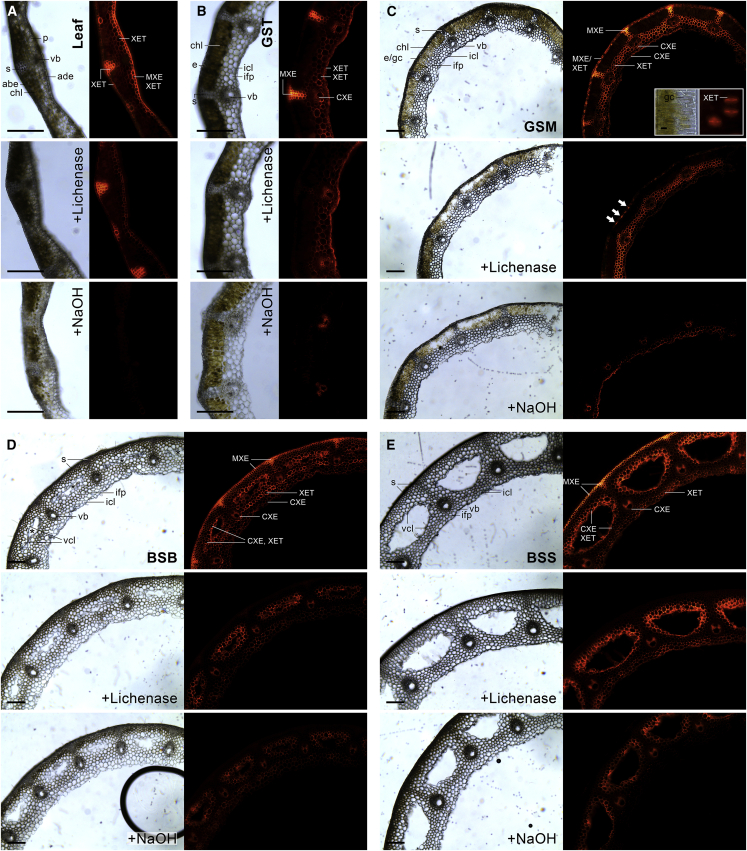
Localization of XET, MXE, and CXE Action and Their Endogenous Donor Substrates in *Equisetum* Leaf and Stem Parts of Different Ages. Bright-field images and corresponding fluorescence showing XXXGol-sulforhodamine incorporation into cell walls of cross-sections. Total fluorescence (top image of each set of three) indicates XET + MXE + CXE products; fluorescence remaining after removal of MLG by lichenase (center image) indicates XET + CXE products; and fluorescence remaining after subsequent NaOH treatment (bottom image) reveals CXE products only. **(A)** Leaf, **(B)** young, and **(C)** middle-aged internode (inset: tangential view of epidermis); **(D)** old internode with vallecular canals (asterisks) starting to appear; **(E)** old submerged internode. abe, abaxial epidermis; ade, adaxial epidermis; e, epidermis; gc, guard cells; icl, inner cortex layer; ifp, interfascicular parenchyma; s, sclerenchyma; vb, vascular bundle; vcl, vallecular canal layer. Plant parts shown are represented in Figure 1A. Scale bars, 250 μm and 25 μm (inset).

**Figure 5:**
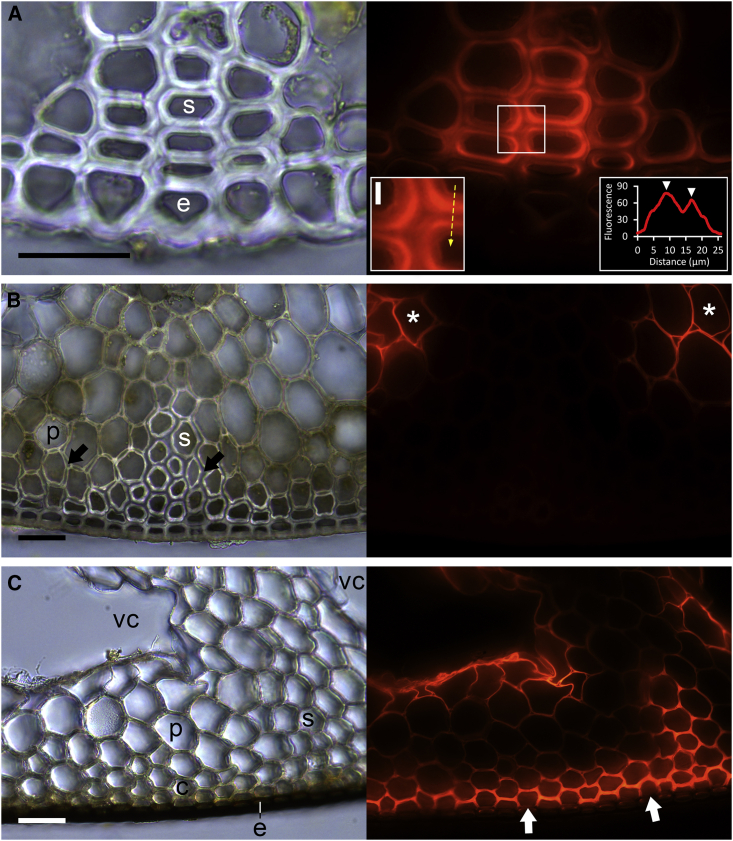
Transglucanase Action in Peripheral Sclerenchyma of *Equisetum* Internodes. Bright-field images and corresponding fluorescence showing XXXGol-sulforhodamine incorporation into cross-sections. **(A)** Young internode. Left inset: central cell-wall region of sclerenchyma cells. Right inset: fluorescence intensity plot over the region marked by the dashed arrow in the left inset; arrowheads mark the intensity maxima in the border zones between primary and secondary cell walls. **(B)** Middle internode. In this specimen, lichenase treatment was applied and was observed to remove MXE products from the central cell-wall region of sclerenchymatous and parenchymatous cells (arrows). **(C)** Old basal internode. Asterisks denote cells surrounding vallecular canals. e, epidermis; p, parenchyma; s, sclerenchyma; vc, vallecular canal. Scale bars, 100 μm and 10 μm (inset).

The following studies localized HTG-specific actions (MXE or CXE). Fluorescent XXXGol-sulforhodamine (acceptor substrate) was incubated with *Equisetum* cross-sections, then fluorescent transglucanase products were sequentially removed, allowing us to separately localize MXE, XET, and CXE products (Figures 4 and 5). To localize MXE action we digested MLG with lichenase, thus specifically removing MXE product (MLG–XXXGol-sulforhodamine; Figures 4 and 5B). A single lichenase treatment removed all detectable MLG (Supplemental Figure 1). After lichenase, NaOH solubilized xyloglucan–XXXGol-sulforhodamine (XET product), leaving only CXE product (cellulose–XXXGol-sulforhodamine; Figure 4).

This strategy revealed MXE action primarily in the sclerenchyma (structural sterome) of *Equisetum* stems (Figure 4B–4E), especially in the central cell-wall regions (Figure 5A). With increasing internode age toward the shoot base, the amount of sclerenchyma with detectable MXE action increased strongly, forming a continuous subepidermal belt by replacing chlorenchymatous tissues (Figure 4B–4E) and paralleling a higher amount of extractable MXE activity (Figure 1A) and MXE action than in younger internodes (Figure 2D). Periclinal walls of the outermost sclerenchyma cells attached to the epidermis lacked MXE action (Figure 5C). Epidermis of leaves (adaxial; Figure 4A) and middle-aged (Figure 4C) but not old (Figure 4D and 4E) internodes showed MXE action, while guard cells in the middle-aged shoot epidermis exhibited XET action (Figure 4C, inset). In general, *Equisetum* epidermal cells are rich in amorphous hydrated silica (Gierlinger et al., 2008), which may add mechanical strength (Epstein, 1999). Occasionally (∼10% of shoots), abundant MXE action was found in the interfascicular parenchyma of young internodes (Supplemental Figure 2). Although this observation was not explored in detail, a plausible interpretation might be that in certain stages of internode development, and before vallecular canals emerge between vascular bundles, HTG is able to act on MLG in parenchyma and introduce links between MLG and xyloglucan (MXE action), potentially providing additional mechanical stability to the shoot. The time window for this MLG modification in parenchyma might be short, explaining its absence in most GSM internodes.

*Equisetum* CXE action occurred in the xylem and cell layers surrounding carinal canals from the stem tip to base but not in leaves (Figure 4 and Supplemental Figure 3). With increasing shoot age, CXE action was additionally detectable in the inner cortex layer (Figure 4C and 4D) and parenchyma surrounding the vallecular canals (Figure 4D and 4E; Figure 5B and 5C). Cell walls of these tissues with abundant CXE action are rich in both xyloglucan and crystalline cellulose (peaking in carinal canal linings; Sørensen et al., 2008, Leroux et al., 2011). Carinal canals form during internode elongation (Browne, 1912) and are involved in water transport (Buchholz, 1921, Xia et al., 1993). In contrast, vallecular canals are interrupted at the nodes, are not connected to the water system, and function as aerenchyma in submerged stems (Krähmer, 2016). However, carinal and vallecular canals have in common that they are highly ordered cavities, their formation is growth-stage-dependent, and they are surrounded by one or two cell layers that exhibit high CXE action. Finding abundant CXE action in certain cell types was surprising, since *Pichia*-produced *Equisetum* HTG (*Ef*HTG) exhibits very much higher CXE activity on alkali-pre-treated filter-paper (type II cellulose) than on plain filter-paper (type I cellulose) (Simmons et al., 2015). The native cellulose in plant cell walls is assumed to be predominately type I. For the endogenous cellulose to serve as a donor substrate at selected sites and times, it must be far more accessible to HTG than is filter-paper. Possible explanations for this observation could be (1) more easily accessible amorphous (or possibly type II) cellulose occurring *in vivo*, (2) some localized covalent modification that exposes portions of cellulose chains in certain microfibrils, e.g., oxidation by hydroxyl radical attack (Fry, 1998), or (3) localized expansin action exposing certain cellulose chains.

The presence of both MXE and XET action (decrease in fluorescence after lichenase; further decrease after NaOH) was only observed in adaxial leaf epidermis, while XET and CXE action co-occurred in tissue surrounding vallecular canals (Figure 4D and 4E).

In charophytic algae (*Chara vulgaris*, *Zygnema circumcarinatum*), grasses (*P. annua*, *Holcus lanatus*), and a dicot (*Alnus glutinosa*), no MXE action was detectable (Supplemental Figures 4 and 5). XET action occurred in most cell walls of algae (Supplemental Figure 4; see also Herburger et al., 2018) and in the epidermis, trichomes, structural tissues, and vascular bundles of grasses (Supplemental Figure 5A–5D) and *Alnus* (Supplemental Figure 5E and 5F). NaOH treatment removed fluorescence completely (Supplemental Figures 4 and 5), indicating absence of CXE action. As expected, xyloglucan endoglucanase treatment yielded the same result as NaOH (Supplemental Figures 6 and 7). Feeding XXXGol–SR to heat-inactivated tissue sections did not yield appreciable fluorescence signals (Supplemental Figure 8), demonstrating that XGOs cannot remain non-covalently bound to the cell wall after thorough washing.

### Bacterial Expansin Strongly Stimulates CXE Activity

**Figure 6:**
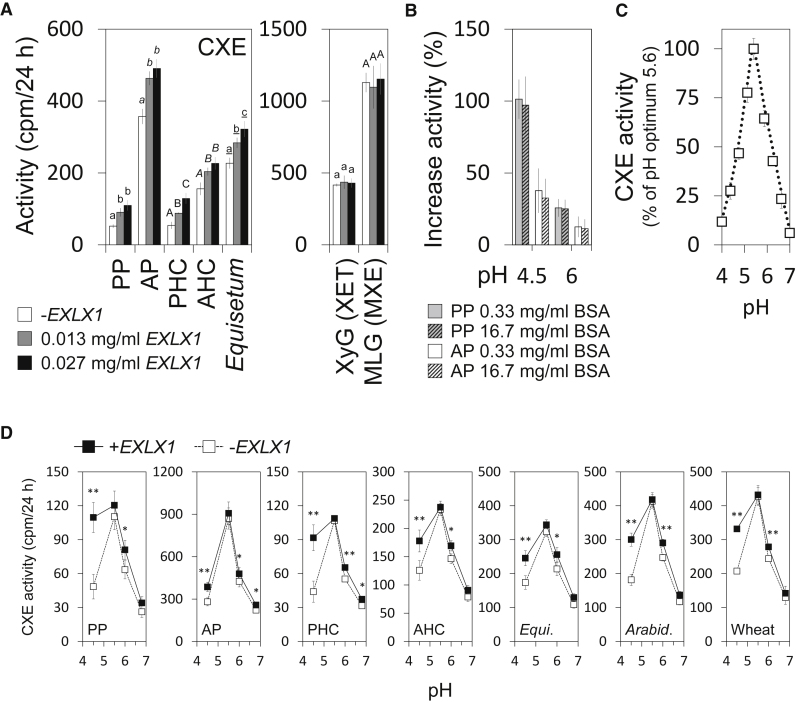
Effect of Bacterial Expansin on CXE Hetero-transglucosylation by *Ef*HTG. **(A)** Left: bacterial expansin (EXLX1) concentration-dependent increase of CXE activity acting on various insoluble cellulosic donor substrates (pH 6; acceptor substrate: [^3^H]XXXGol); significant differences (*p* < 0.05) between activities at different concentrations are marked by letters; *n* = 3 ± SD. Right: analogous assays testing the soluble donor substrates xyloglucan (XET activity) or MLG (MXE activity; pH 6); *n* = 3 ± SD. **(B)** Effect of bacterial expansin (0.013 mg/ml) on CXE activity in the presence of different BSA concentrations; *n* = 3 ± SD. **(C)** pH dependence of CXE activity on AP; *n* = 3 ± SD. **(D)** Increase of CXE activity due to bacterial expansin (0.013 mg/ml) at different pH values. Significant differences between assays with (+EXLX1) and without bacterial expansin (−EXLX) at a given pH are marked by asterisks (∗*p* < 0.05, ∗∗*p* < 0.01); *n* = 3 ± SD. PP, plain Whatman no. 1 paper; AP, alkali-treated Whatman no. 1 paper; PHC, plain paper handkerchief; AHC, alkali-treated paper handkerchief; *Equi*., *Equisetum* stem cellulose; *Arabid*., *Arabidopsis* stem cellulose; Wheat, wheat stem cellulose.

The protein HTG exhibits both MXE and CXE activities, yet the above work (Figure 2) shows that the MXE:CXE action ratio varies between different tissues and organs of *Equisetum*. In particular, some tissues (e.g., sclerenchyma) show almost no CXE action despite possessing both the required substrates (cellulose and xyloglucan) and exhibiting high MXE action (Figures 4 and 5). Likewise, Figure 1B shows that the CXE:MXE activity ratio in crude extracts from roots is consistently ∼3.5-fold higher than in those from internodes and rhizomes. We therefore hypothesized that “high CXE” tissues and organs contain a factor that renders cellulose more accessible to HTG. One such factor could be expansin, which loosens cellulose and cellulose–xyloglucan superstructures (Cosgrove, 2000). To test this, we produced a bacterial expansin in *Escherichia coli* (Supplemental Figure 9) and added it to CXE assays (Figure 6A, 6B, and 6D). We choose the bacterial expansin EXLX1 (from *Bacillus subtilis*) because it is particularly well characterized and shows a similar structure and very similar wall extension activities to those of plant expansins (Kerff et al., 2008, Georgelis et al., 2011), which remain difficult to be produced heterologously (Yactayo-Chang et al., 2016). When bacterial expansin was added to various cellulosic donor substrates (pre-treated with NaOH or not), CXE activity was stimulated with increasing EXLX1 concentrations at pH 6 (Figure 6A). In contrast, bacterial expansin had no stimulating effect when added to XET or MXE activity assays (Figure 6A). Bacterial expansin itself does not possess transglucanase activity (data not shown).

Compared with plant α- and β-expansins, bacterial expansin has a broader pH optimum with activities (i.e., creep of plant cell walls) from pH ∼4.5 to ∼11.5 (Georgelis et al., 2011). *Ef*HTG shows appreciable activities between pH ∼4 and 7 (Figure 6C), allowing us to test the stimulating effect of bacterial expansin at a broad pH range (Figure 6D). Interestingly, bacterial expansin-mediated CXE activity stimulation was consistently highest at low pH (4.5), insignificant at pH 5.5 (*Ef*HTG's pH optimum; Figure 6C), and moderately high at higher pH values (pH 6 and 6.8; Figure 6C).

CXE assays are routinely conducted in the presence of 0.33 mg/ml bovine serum albumin (BSA), which seems to prevent the immobilization of HTG on the cellulose. Interestingly, bacterial expansin at only 0.013 mg/ml was able to enhance CXE activity in the presence of this 25-fold excess of BSA, indicating that the expansin was not merely exerting a general “inert protein” effect. Furthermore, bacterial expansin was still able to enhance CXE activity even in the presence of 16.7 mg/ml BSA, a 1250-fold excess of inert protein (Figure 6B), confirming that a unique activity of expansin was responsible for its ability to enhance CXE activity. These results together show that expansin strongly synergizes with CXE activity, and thus potentially CXE action *in vivo*, in a pH-dependent manner.

## Discussion

### Cellulose–Xyloglucan Covalent Linkages Formed *In Situ*

Here, we show that endogenous cellulose can be covalently attached to xyloglucan oligosaccharides (XGOs) in living *Equisetum* tissues, and thus provide evidence that native cellulose can undergo enzymic covalent modifications *in muro* other than hydrolysis. Thus, this report is distinct from all previous studies on cellulose hetero-transglucanases, as they measured the enzymes' activity (i.e., *in vitro*, in a test tube), but not its action (i.e., *in situ* on its natural donor substrates). Furthermore, we found that MLG is covalently grafted onto XGOs in structural tissues. Both these reactions (CXE and MXE action, respectively) are catalyzed by a single enzyme, HTG (Simmons et al., 2015).

Using newly developed techniques, we have quantified and visualized the distribution of CXE and MXE action within *Equisetum* organs. These findings indicate that current structural models of plant cell walls—as presented in biology textbooks (Kadereit et al., 2013)—oversimplify cellulose–hemicellulose interactions. Proof of cellulose–xyloglucan covalent bond formation in cell walls is interesting in the light of emerging primary cell-wall models based on atomic-force microscopy, field-emission scanning electron microscopy, and solid-state nuclear magnetic resonance data, which suggest the formation of “biomechanical hotspots” in the cell wall, where amorphous cellulose and xyloglucan intertwine (Cosgrove, 2018). Digesting these load-bearing junctions with specific enzymes induced cell-wall softening and creep/extension (Park and Cosgrove, 2012). The origin of such cellulose–xyloglucan junctions is unclear, but it is possible that they are formed enzymically by hetero-transglucosylation (Zheng et al., 2018).

### Synergy of *Ef*HTG with Expansin

This idea is particularly interesting in the light of our finding that *in vitro* CXE activity is strongly increased in the presence of an expansin. Expansins are considered to induce their effect by acting on proposed “biomechanical hotspots” (Wang et al., 2013) and cause an irreversible wall deformation (creep) in response to mechanical tension (e.g., turgor) by inducing a viscoelastic fluid behavior of the cell wall, which stops upon inactivation of expansin (Takahashi et al., 2006). Since expansin and cellulose hetero-transglucosylation can act together, it is plausible that this synergy might provide certain plant cells and tissues with an additional tool to control the viscoelastic/plastic behavior of their cell walls: expansin action facilitates turgor-driven cell expansion, while co-occurring cellulose:xyloglucan hetero-transglucanase action may continuously help to re-establish stabilizing tethers between cellulose microfibrils. Once growth ceases, xyloglucan–cellulose tethering may help to stabilize the wall. According to the acid growth theory, auxin-mediated acidification of the apoplast down to pH 5 and below drives cell expansion (Hager et al., 1971, Arsuffi and Braybrook, 2017). Intriguingly, at pH values lower than the optimum of *Ef*HTG (i.e., pH < 5.6) and which are in the apoplastic range, the expansin-mediated stimulation of CXE activity was consistently highest for all cellulosic substrates (Figure 6D). Our transcriptomic data confirm that the genus *Equisetum* expresses expansin genes in shoot apices, sterile leaves, and branches, and in expanding and mature stems. *EXPANSIN A* mRNA predominates and *EXPANSIN B* gene expression was only found in *Equisetum hyemale* and *Equisetum diffusum*, the closest related species to *Equisetum fluviatile* (Supplemental Figure 10).

It is surprising that in several independent experiments and using different cellulosic substrates, the stimulation of CXE activity by bacterial expansin is consistently higher at low pH than it is at high pH, even though bacterial expansin was shown to be active over a broad pH range (Georgelis et al., 2011). It could be speculated that HTG, an enzyme working on cellulose, itself exhibits expansin-like activity with a narrow pH optimum around 5.5, at which pH its CXE activity cannot be elevated much further by addition of bacterial expansin. Further studies may test HTG for expansin activity, e.g., by evaluating its weakening effect on pure cellulose filter-paper in the absence of any transglucanase acceptor substrates (xyloglucan or XGOs) so that only expansin-like but not transglucanase activity could be exerted by the HTG. Interestingly, CXE action is measurable in very young, rapidly growing internodes, but increases with age and peaks in established internodes and cells which ceased growth. The role of expansin in cell elongation is widely accepted. On the other hand, strong expansin overexpression does not necessarily increase the extensibility or accelerates growth of plants, as one might have expected, but can even reduce their growth and size (Brummell et al., 1999, Rochange et al., 2001). Even though not further explored in detail, it cannot be excluded that increased expansin action significantly alters the action rate of cell-wall remodeling enzymes such as HTG that may not only have their role in cell-wall growth.

While our results showed a stimulating effect of expansin on a transglucanase, several previous studies have implicated expansin as a cellulase synergist to enhance cellulose/biomass hydrolysis (Kerff et al., 2008, Kim et al., 2013, Lin et al., 2013, Georgelis et al., 2015, Martinez-Anaya, 2016). Most of these studies used BSA as a control, which sometimes exhibited synergistic effects similar to those of added expansin (e.g., Kim et al., 2013), while others found a considerably higher stimulation by expansin. For example, Lin et al. (2013) reported that the presence of bacterial expansin EXLX1 at 0.02 mg/ml increased the release of reducing sugars by cellulase from untreated wheat straw by up to ∼60%, about three times higher than does BSA. In general, the expansin/cellulase synergism was strongest with small cellulase loadings where expansin might block non-specific substrate sites or reduce non-productive cellulase binding, possibly a beneficial surfactant effect similar to that exerted by BSA or non-enzymic proteins present in cattle saliva (Kerff et al., 2008, Seki et al., 2015a).

Comparable studies on plant expansins are rare, as producing them heterologously remains challenging (Yactayo-Chang et al., 2016). However, Seki et al. (2015b) reported that some *Oryza sativa* α- and β-expansins (expressed in *E*. *coli* in small quantities) augmented the ability of cellulase to release glucose from suspended crystalline cellulose slightly more effectively than did BSA (0.15% versus 0.13% total cellulose hydrolysis; Seki et al., 2015b). This suggests that both bacterial and plant expansins can act as synergists for cellulose-active enzymes that exceeds the effect of BSA. In contrast to the results reported for the effect of bacterial (EXLX1) and plant expansins on cellulase activity, the stimulating effect of bacterial expansin EXLX1 on *Ef*HTG's CXE activity occurs even in the presence of a 1250-fold excess of BSA (Figure 6B).

### Possible Role of HTG in Structural Tissues

**Figure 7:**
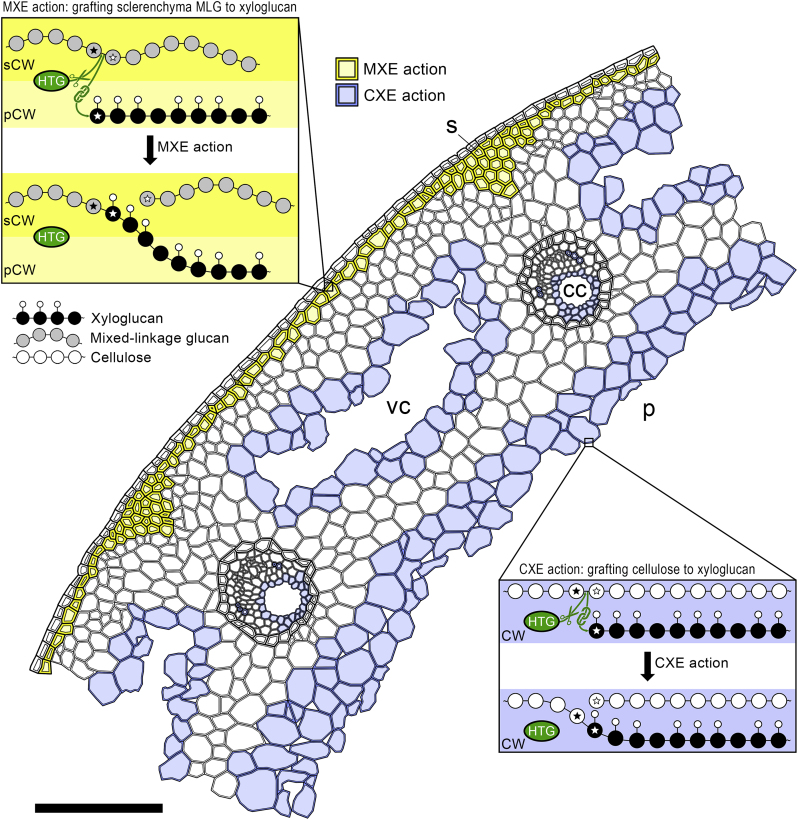
Proposed Roles and Sites of Unique HTG Transglucanase Actions in an *Equisetum* Stem. Scheme represents a cross-section, where MXE action (yellow) predominates in sclerenchyma cell walls and CXE action (blue) in cell walls bordering vallecular and carinal canals and the central pith. Insets illustrate MXE action (connecting primary and secondary cell walls) and CXE action (grafting cellulose onto xyloglucan). Monomers involved in breakage and reformation of bonds are marked by stars. cc, carinal canal; CW, cell wall; p, pith; pCW, primary cell wall; s, sclerenchyma; sCW, secondary cell wall; vc vallecular canal. Scale bar, 250 μm.

While XET action has been studied intensively and shown to be involved in numerous physiological processes (Franková and Fry, 2013), the significance of hetero-transglucosylation is not well understood. The amount of CXE action in *Equisetum* stems was appreciable, amounting to an incorporation of up to ∼0.5 ng XXXGol/g fresh weight (FW)/24 h into cellulose, which corresponds to ∼60% of XET action (0.8 ng XXXGol/g FW/24 h into xyloglucan; Figure 2E). However, this probably underestimates the actual CXE action, because the amount of acceptor substrate supplied in feeding experiments (50 kBq/750 μl ≈ 0. 1 μM [^3^H]XXXGol) was much lower than the optimum concentration (*K*_M_ of CXE activity = 2.7 μM XXXGol; Simmons et al., 2015). CXE action increases with age and peaks in established internodes that have ceased growing, where it might play a strengthening role. Intriguingly, CXE action is abundant in cells surrounding cavities (carinal and vallecular canal, cavity in stem center; Figure 7); bond formation between cellulose and xyloglucan in these cells, which had ceased growth and division, might support their walls, thus preventing local injuries due to fluctuating pressure (water flow in carinal canals) or shearing forces caused by mechanical impact (e.g., strain on vallecular cannel border cells due to stem expansion). If true, further studies may elucidate whether CXE action provides strength by hetero-polymer formation and/or affects the crystallinity of cellulose. The latter is crucial for defining the cell wall's mechanical properties; for example, the plant's lodging resistance is reduced by a higher cellulose crystallinity (Li et al. 2013). As shown for grasses such as *Miscanthus* sp. and rice, arabinose-rich hemicelluloses (arabinoxylans) reduce the cellulose crystallinity by interacting non-covalently with the β-1,4-glucan chains in amorphous regions of cellulose microfibrils (Li et al., 2013, Li et al., 2015). HTG does not utilize xylans as donor or acceptor substrates (Fry et al., 2008b, Simmons et al., 2015); however, xyloglucan might exercise similar effects (Wang et al. 2016), and it is possible that its covalent bonding to cellulose affects cellulose crystallinity and, thus, plant stability.

Hetero-transglucosylation being important for tissue stability is further supported by extensive MXE action in sclerenchyma (Figures 5A and 7), on which the stiffness and strength of *Equisetum* stems mainly relies (Speck et al., 1998), while other structural tissues such as endodermal layers surrounding vascular bundles are of minor importance for stem stability (Spatz and Emmans, 2004). Sclerenchyma secondary walls are particularly rich in MLG (Sørensen et al., 2008) while xyloglucan peaks in primary cell walls (Leroux et al., 2011). We found the highest MXE action in the central wall area of sclerenchyma cells, with signals decreasing toward both the middle lamella and the plasma membrane (Figure 5A). This suggests that HTG action predominates in the border zone between primary and secondary cell wall and covalently bonds the MLG-rich secondary wall to the xyloglucan-rich primary wall (Figure 7). This could contribute to the crucial strengthening role of sclerenchyma in *Equisetum*. In agreement, young stem tips, rhizomes, and roots exhibit only very low extractable MXE activity and *in situ* MXE action, maybe because of a lesser need for mechanical stability, since these organs are not the structural fundament of a stem or are stabilized by the substrate and/or soil water, respectively, and are thus exposed to smaller mechanical forces.

MXE action in native plant cell walls is interesting in the light of a recently discovered MXE-predominant hetero-transglucanase (*Bd*XTH8) in the grass *Brachypodium* (Fan et al., 2018). However, neither we (Figure 2D and Supplemental Figure 5A–5D) nor previous workers (Mohler et al., 2013) detected *in-vivo* MLG–XGO covalent bonding in grasses, suggesting that MXE action is not prevalent in MLG-rich angiosperms. This “missing reaction” could be conferred on MLG-rich poalean crops by equipping them with hetero-transglucanase action by genetic insertion of *EfHTG*, which could increase plants' tissue strength and therefore their resistance to mechanical stress. This would have high potential to decrease crop failure caused by wind or water lodging. The flowering plants included in the present study (*P. annua*, *H. lanatus*, *A. glutinosa*) lacked appreciable extractable MXE or CXE activity in *in vitro* assays (see also Fry et al., 2008b) and thus served as control groups in our *in situ* experiments testing for MXE and CXE action (Figure 2B and Supplemental Figure 5). In contrast, earlier studies suggest the presence of MXE activity (*in vitro*) in extracts from charophyte green algae (Fry et al., 2008b, Franková and Fry, 2011a, Herburger et al., 2018). However, our *in-situ* visualization studies did not find MXE action in algal cell walls (Supplemental Figure 4). This might be because the *in-vitro* studies used non-intrinsic commercial grass MLG as donor substrates, which may not be present in sufficient concentrations in the algal cell wall (Herburger et al., 2018) or because the fed XXXGol-sulforhodamine was attached to an “ancestral” algal β-glucan, which cannot be removed by commercial lichenase.

The absence of CXE action in the charophytes and non-fern vascular plants tested implies that this reaction might not be universal among land plants. On the other hand, its substrates (cellulose + xyloglucan) occur in all land plants, and *Arabidopsis* produces a hetero-transglucanase (*At*XTH3) which, among other reactions, covalently grafts cellulose onto XGOs *in vitro* (Shinohara et al., 2017). Similar enzymes were found in barley and nasturtium (Hrmova et al., 2007, Stratilová et al., 2019). This suggests that CXE action might occur in both monocots and dicots, but has been overlooked so far owing to the unavailability of sensitive methods to quantify and localize it. Our novel methods provide the wherewithal for broader screening of the plant kingdom for CXE and MXE action in plant tissues. The methods presented here use exogenous labeled oligosaccharides (XGOs) to test for hetero-polymer formation; however, future studies may provide evidence for the *in planta* formation of MLG–xyloglucan and cellulose–xyloglucan hetero-polymers.

## Methods

### Plant Sources and Materials

*E*. *fluviatile* (Figure 1A) was grown in a pond outside the Institute of Molecular Plant Sciences of the University of Edinburgh (Edinburgh, UK) or collected from the Pentland Hills (Edinburgh). Samples were taken in late January (late winter) and August 2017 (late summer) and April 2018 (mid spring) and analyzed immediately after collection. *P. annua* and *H. lanatus* plants and *A. glutinosa* twigs were collected from a meadow in Edinburgh. Axenic cultures of *Z. circumcarinatum* and *C. vulgaris* were grown in Bold's basal medium (Bischoff and Bold, 1963) for 1.5 or 3 months, respectively. *Tamarindus indica* seed xyloglucan was a gift from Dainippon Pharmaceutical (Osaka, Japan); barley MLG (β-glucan; medium and high viscosity), lichenase (from *B. subtilis*), xyloglucan-inactive cellulase (from *Aspergillus niger*), and cellobiohydrolase (from *Trichoderma longibrachiatum*) were purchased from Megazyme. Xyloglucan endoglucanase was a gift from Novo Nordisk (Bagsværd, Denmark; Pauly et al., 1999). [^3^H]XXXGol (for xyloglucan-oligosaccharide [XGO] nomenclature see Fry et al., 1993) and XXXGol-sulforhodamine, prepared as described previously (Hetherington and Fry, 1993, Miller et al., 2007), were from EDIPOS (http://fry.bio.ed.ac.uk//edipos.html). Thin-layer chromatography was performed on silica-gel 60 plates (Merck). Other chemicals were purchased mainly from Sigma-Aldrich (Poole, UK).

### Heterologous Enzyme Production and Enzyme Extraction from Plant Tissues

Heterologous production of *Ef*HTG and *Ef*XTH-H using a recombinant pPICZaA vector system in *Pichia pastoris* strain SMD1168H was as described elsewhere (Simmons et al., 2015). Both these *Equisetum* enzymes are highly acidic (p*I* of *Ef*HTG and *Ef*XTH-H ≈ 4.1 and 4.6, respectively); *Ef*XTH-H has XET but very little MXE and CXE activity (Holland et al., 2020). Enzyme extraction from *Equisetum* followed the protocol of Fry et al., 2008a, Fry et al., 2008b. In brief, ∼0.5–1.5 g of tissue was ground in ice-cold extraction buffer (5 ml/g fresh weight) containing 0.3 M succinate (Na^+^, pH 5.5) and 3% (w/v) polyvinylpolypyrrolidone, and the supernatant was either used immediately in assays of XET, MXE, and CXE activity or stored at −80°C until processed. *H. lanatus* crude protein extraction buffer contained 0.3 M succinate (Na^+^, pH 5.5), 10 mM CaCl_2_, 20 mM ascorbic acid, and 15% glycerol. *H. lanatus* proteins were precipitated with ammonium sulfate at 40% saturation.

The gene *yoaJ*, encoding bacterial expansin (EXLX1) from *B. subtilis* (Kerff et al., 2008), was expressed in *E*. *coli* JM109, via the plasmid pSB1C3 with lac promoter. Bacterial expansin EXLX1 was produced in Luria–Bertani medium (10 g/l tryptone, 5 g/l yeast extract, 10 g/l NaCl) at 37°C, cultures being induced at mid-exponential phase (OD ≈ 0.5) with 0.4 mM isopropyl β-d-1-thiogalactoside for 16 h. Bacterial expansin-producing (pSB1C3 *yoaJ*) and non-producing (pSB1C3 empty) *E*. *coli* cells were harvested by centrifugation, suspended in ice-cold phosphate-buffered saline (PBS) (15 ml/g cells) containing 12.5% (v/v) glycerol, and lysed via sonication. Cell debris was spun down and the supernatant either used immediately or frozen at –80°C. The presence of bacterial expansin (EXLX1; ∼23 kDa) in transformants was confirmed by SDS–PAGE followed by Coomassie blue staining and quantified by reference to a BSA concentration gradient and ImageJ. Available *Equisetum* transcriptomes (Frank et al., 2015, Simmons et al., 2015; www.onekp.com) were mined for *EXPANSIN A* and *B* sequences.

### Preparation of Radiolabeled Xyloglucan

[^3^H]Xyloglucan was produced by XTH-catalyzed grafting of tamarind xyloglucan (∼10^6^ Da) to [^3^H]XXXGol. [^3^H]XXXGol (0.5 MBq) was added to 200 μl of 0.5% (w/v) xyloglucan in 62.5 mM citrate (Na^+^, pH 6.3), mixed with 100 μl of *H. lanatus* extract, and incubated at 20°C for 48 h to catalyze xyloglucan:[^3^H]XXXGol transglucosylation. Proteins were denatured (1 h, 100°C) and the supernatant was dialyzed against running tap water (64 h), dried, and repeatedly washed in 75% ethanol until the supernatant lacked radioactivity ([^3^H]XXXGol). Precipitated [^3^H]xyloglucan was redried and redissolved in 0.5% chlorobutanol, and its lack of contamination by unreacted [^3^H]XXXGol was confirmed by paper chromatography (*R*_F_ 0) and scintillation counting. Its specific radioactivity was ∼200 Bq/μg.

### Assay of Radioactivity

^3^H in aqueous solutions was quantified by scintillation counting in ScintiSafe 3 scintillation fluid (Fisher Scientific, Loughborough, UK); ^3^H bound to dried papers by scintillation counting in GoldStar “O” scintillation fluid (Meridian, Chesterfield, UK).

### *In-Vitro* Radiochemical Assay of XET, MXE, and CXE Activities

XET and MXE activities were assayed with 5 μl of filtrate from *Pichia* cultures expressing *Ef*HTG or *Ef*XTH-H, or *Equisetum* protein extracts, in 20 μl total volume containing (final concentrations) 0.1 M succinate (Na^+^, pH 5.5), 0.1% (w/v) BSA, 0.4–1.0 kBq acceptor substrate ([^3^H]XXXGol or [^3^H]xyloglucan), and 0.5% (w/v) donor substrate (xyloglucan or MLG for XET or MXE activity, respectively). For testing CXE activity (Simmons et al., 2015), 20 mg of Whatman no. 1 paper (insoluble donor; pre-treated with 6 M NaOH, thus cellulose II) was soaked with 20 μl of the above reaction mixture omitting soluble donor substrates. After 24 h of incubation at 22°C, transglycosylation reactions were stopped by addition of 6 μl of 90% formic acid. XET and MXE products were dried on Whatman no. 3 paper, washed in running tap water overnight, and quantified by scintillation counting. CXE reactions were stopped by addition of 30% formic acid, then the cellulose was washed sequentially in 6 M NaOH for 12 h at 20°C, 6 M NaOH for 1 h at 100°C, and running tap water overnight, and assayed for bound ^3^H in “Gold Star” scintillant. Control groups in XET, MXE, and CXE assays contained heat-inactivated enzymes or lacked enzymes. The amount of detectable ^3^H in control groups (13–18 cpm) was comparable with the signal obtained with plain paper (no sample on it: 10–13 cpm) and was subtracted as “background ^3^H” from experimental groups.

For estimation of the counting efficiency of paper-bound ^3^H products, the papers from some of the above assays were recovered from the scintillation fluid, rinsed in acetone, dried, and incubated in 2 M TFA at 120°C for 1 h; after removal of TFA *in vacuo*, the solubilized products (redissolved in water) were assayed for ^3^H by scintillation counting in OptiPhase HiSafe 3 scintillant with quench correction (Supplemental Figure 11).

To profile diagnostic fingerprints of *in vitro*-formed XET, MXE, and CXE products, we prepared polymer–[^3^H]XXXGol conjugates enzymically *in vitro*. After digestion with commercial enzymes, the products were analyzed by thin-layer chromatography (two ascents in butan-1-ol/acetic acid/water [2:1:1]) and profiled on a radioisotope scanner (AR2000; LabLogic, Sheffield, UK).

### Assaying the Effect of Bacterial Expansin on CXE Activity

Cellulosic substrates were Whatman no. 1 paper and paper handkerchiefs (Tempo Original; Svenska Cellulosa) (both untreated or 6 M NaOH pre-treated), *Equisetum*, *Arabidopsis*, and wheat cellulose (isolated from stems; Fry, 2000). Cellulosic substrates (20 mg) were soaked in 20 μl of PBS containing 400–800 ng of bacterial expansin EXLX1, followed by addition of 10 μl of solution A (5 μl of *Ef*HTG and 1 kBq [^3^H]XXXGol in 0.45 M succinate [Na^+^] and routinely 0.1% BSA [w/v]). The final pH (4.5–6.8) of the reaction mixture was achieved by use of an appropriate pH in the solution A such that when 10 μl was mixed with 20 μl of PBS, the desired pH was obtained. In some experiments, cellulosic donor substrates were pre-treated with bacterial expansin for up to 4 h or replaced by 20 μl of a soluble donor substrate (xyloglucan or MLG).

### *In Situ* Visualization of Transglucanase Action

*Equisetum* stems from different shoot heights (tip, middle, base) representing a gradient of increasing tissue age, and leaves and stems/twigs of *P. annua*, *H. lanatus*, and *A. glutinosa* were hand-sectioned with a razor blade to a thickness of ∼200 μm and incubated in 150 μl of 25 mM succinate (Na^+^, pH 5.5) containing ∼5 μM XXXGol-sulforhodamine (Vissenberg et al., 2000) for 2–4 h. Two charophyte green algae (*C. vulgaris*, *Z. circumcarinatum*) were incubated without prior sectioning. Non-incorporated XXXGol-sulforhodamine was removed by washing in ethanol/formic acid/water (6:0.4:4 [v/v/v]) for 10 min and in aqueous 5% (v/v) formic acid overnight. After rinsing in water, sections were examined with a Leica DM2000 LED microscope equipped with a Leica DFC7000 T camera and Leica EL6000 external light source. Incorporated sulforhodamine was visualized with a GFP filter cube (BP 470/40, emission BP 525/50). As controls, XXXGol-sulforhodamine was omitted or sections were boiled for 5 min before addition of XXXGol-sulforhodamine (Supplemental Figure 8). Images were taken with LAS X software and assembled in Adobe Photoshop CC and ImageJ (Schindelin et al., 2012) Minimal adjustments to contrast were applied equally across entire image plates.

### Separating XET, MXE, and CXE Action *In Situ* by Enzymic Digestion and Alkali Treatment

*Equisetum* plant parts, the grasses *P. annua* and *H. lanatus* (leaves, stems), the dicot *A. glutinosa* (leaves, twigs), and charophyte green algae were fed with XXXGol-sulforhodamine and imaged as described in the previous paragraph. Subsequently, sections were recovered from slides, digested with lichenase (2.5 units/50 μl, 25 mM citrate [Na^+^, pH 6.5] or 1:1:98 pyridine/acetic acid/water [pH 4.7]) for 2 × 6 h, imaged again, then incubated with 6 M NaOH at 37°C for 12 h and imaged again. Some of the sections were subjected to digestion with xyloglucan endoglucanase (50 μl; 0.5% in pyridine/acetic acid/water [1:1:98], pH 4.7) instead of lichenase. Substrate specificity of lichenase and xyloglucan endoglucanase was tested using a standard viscometric assay (Fry, 1998). To test whether a proportion of MLG in the sections is not accessible for enzymic hydrolysis, we digested some sections (20 mg) with lichenase (2 × 6 h, in pyridine/acetic acid/water [1:1:98], pH 4.7), then rinsed in 72% EtOH, and the supernatant was assayed for MLG oligosaccharides separable by thin-layer chromatography in butan-1-ol/acetic acid/water (2:1:1; three ascents). The MLG oligosaccharide pattern extractable from sections treated with lichenase was compared with the pattern obtained by lichenase-digestion hemicelluloses that had been extracted from sections with 6 M NaOH (3 × 1 day, 37°C).

### Quantifying XET, MXE, and CXE Action in Native Plant Cell Walls

*Equisetum* internodes from different shoot parts, rhizomes, and roots of four individual plants (*n* = 4) were cut into ∼300-μm slices and ∼250 mg of sliced tissue was immediately incubated with 500 μl of 25 mM succinate (Na^+^, pH 5.5) containing 50 kBq [^3^H]XXXGol and 0.1% (w/v) chlorobutanol under gentle shaking for 24 h at room temperature. Reactions were stopped with 600 μl of 0.5% formic acid in 96% EtOH and the tissue was washed in an ethanol series (90%, 80%, 70%, 60%, 50%, 30%) and water until the supernatant lacked detectable ^3^H. Hemicelluloses were extracted from the resultant alcohol-insoluble residue with 6 M NaOH (4 × 24 h at 37°C), neutralized with acetic acid, and dialyzed for 4 days against water. Both the precipitated material (“hemicellulose A”) and the water-soluble fraction (“hemicellulose B”) were freeze-dried and, after pooling, digested with lichenase (2.5 units/250 μl, pH 6.5) and then xyloglucan endoglucanase (250 μl of 0.1%, pH 4.7). Lichenase releases thin-layer chromatography-mobile Glc_2_•[^3^H]XXXGol, which is diagnostic of MXE products (MLG–[^3^H]XXXGol), while xyloglucan endoglucanase releases [^3^H]XXXGol from the remaining XET products (xyloglucan–[^3^H]XXXGol; Mohler et al., 2013). Any remaining undigested polysaccharides (e.g., pectins and mannans) were precipitated with 75% EtOH and small digestion products in the supernatant were analyzed by scintillation counting. The NaOH-insoluble “cellulosic” fraction was boiled in 6 M NaOH for 1 h to extract any remaining traces of hemicelluloses. The cellulosic pellet was then neutralized, dialyzed against water, dried, and subjected to a series of enzymic digestions (each 4 h at 37°C; reaction stopped with 75% EtOH): lichenase (5 units/250 μl, pH 6.5) to digest any MLG–[^3^H]XXXGol still left after NaOH treatments, 3× xyloglucan-inactive cellulase (5 units/250 μl, pH 4.7), 2× xyloglucan-inactive cellulase combined with cellobiohydrolase (5 units [cellulase] and 5 milliunits [cellobiohydrolase] 250 μl, pH 4.7), and xyloglucan-inactive cellulase (5 units/250 μl, pH 4.7) again. ^3^H released into the supernatant after each enzymic digestion step was quantified by scintillation counting. Finally, the cellulosic pellet left after these enzymic digestions was washed with water and treated with 2 M TFA at 120°C for 1 h and the released ^3^H quantified. For control groups, [^3^H]XXXGol was omitted or heat-inactivated sections were used. Furthermore, *P. annua* stem and leaf sections, which show extractable XET but not MXE and CXE activities as verified by our *in vitro* radiochemical assay, were assayed as biological control groups.

For the NaOH-insoluble cellulose described in Figure 2, boiling NaOH was omitted; instead, after treatment with 6 M NaOH at 37°C, the α-cellulose residue was treated with 2 M TFA (1 h at 120°C), hydrolyzing any remaining hemicelluloses, and the TFA-resistant ^3^H in the pellet was considered to be CXE products (cellulose–[^3^H]XXXGol) very firmly trapped within microfibrils. After scintillation counting, this residue was recovered from scintillation fluid by acetone washing and digested with a set of enzymes (as listed above; 25 units of each enzyme except for cellobiohydrolase, which was 25 mU). Each enzyme digest was subjected to thin-layer chromatography and the characteristic fingerprints were profiled by scintillation counting. Sugar markers were stained by thymol–H_2_SO_4_ stain (Franková and Fry, 2011b). The CXE products formed in the tissues were matched to those obtained from authentic *in vitro* generated XET, MXE, and CXE products (Figure 3). GXXXGol and GGXXXGol were produced and characterized as described by Simmons and Fry (2017). GGGXXXGol and GGGGXXXGol were produced by partial digestion of 1 mg (4.1 kBq) *in vitro* prepared cellulose–[^3^H]XXXGol conjugate with 10 units xyloglucan-inactive cellulase in 0.2 ml of buffer (pyridine/acetic acid/water [1:1:98], pH 4.7, incubation for 1–3 h at 40°C).

### Statistical Evaluation of Data

All experiments were carried out with two to five independent replicates. Data are represented by their means and standard deviations. Statistically significant differences between groups were determined by one-way analysis of variance followed by Tukey's post hoc test or by a standard *t*-test (Origin 8.5).

## Funding

We thank the UK 10.13039/501100000268Biotechnology and Biological Sciences Research Council (BBSRC; BB/N002458/1) for funding. K.H. thanks the 10.13039/100008398Villum Foundation (project TIPorNOT 00023089) for financial support during manuscript preparation.

## Author Contributions

S.C.F., L.F. and K.H. planned and designed the study; K.H. performed most of the experiments, M.P. synthesized and assayed the [^3^H]xyloglucan; M.V.-O. and C.E.F. produced bacterial expansin (EXLX1); J.W.L. performed some of the expansin assays; F.M. helped with transcriptome sequencing and gene cloning, A.D.H. with recombinant *Ef*HTG procuction. K.H., S.C.F., and L.F. analyzed the data; K.H. and L.F. prepared the figures; K.H. drafted the manuscript; S.C.F. and L.F. edited the manuscript; all authors approved the manuscript.

## Supplemental Information

Document S1. Supplemental Figures 1–11 and Supplemental References

Document S2. Article plus Supplemental Information
