# Supplementary material for: Hetero-trans-β-Glucanase Produces Cellulose–Xyloglucan Covalent Bonds in the Cell Walls of Structural Plant Tissues and Is Stimulated by Expansin
Source: Mol Plant. 2020 Jul 6;13(7):1047–62. doi: 10.1016/j.molp.2020.04.011 (PMC7339142; doi:10.1016/j.molp.2020.04.011)
Supplement: Document S1. Supplemental Figures 1–11 and Supplemental References [file mmc1.pdf]

Molecular Plant, Volume *13*

Supplemental Information

Hetero-trans-β-Glucanase Produces Cellulose–Xyloglucan Covalent Bonds in the Cell Walls of Structural Plant Tissues and Is Stimulated by Expansin

Klaus Herburger, Lenka Franková, Martina Pičmanová, Jia Wooi Loh, Marcos Valenzuela-Ortega, Frank Meulewaeter, Andrew D. Hudson, Christopher E. French, and Stephen C. Fry

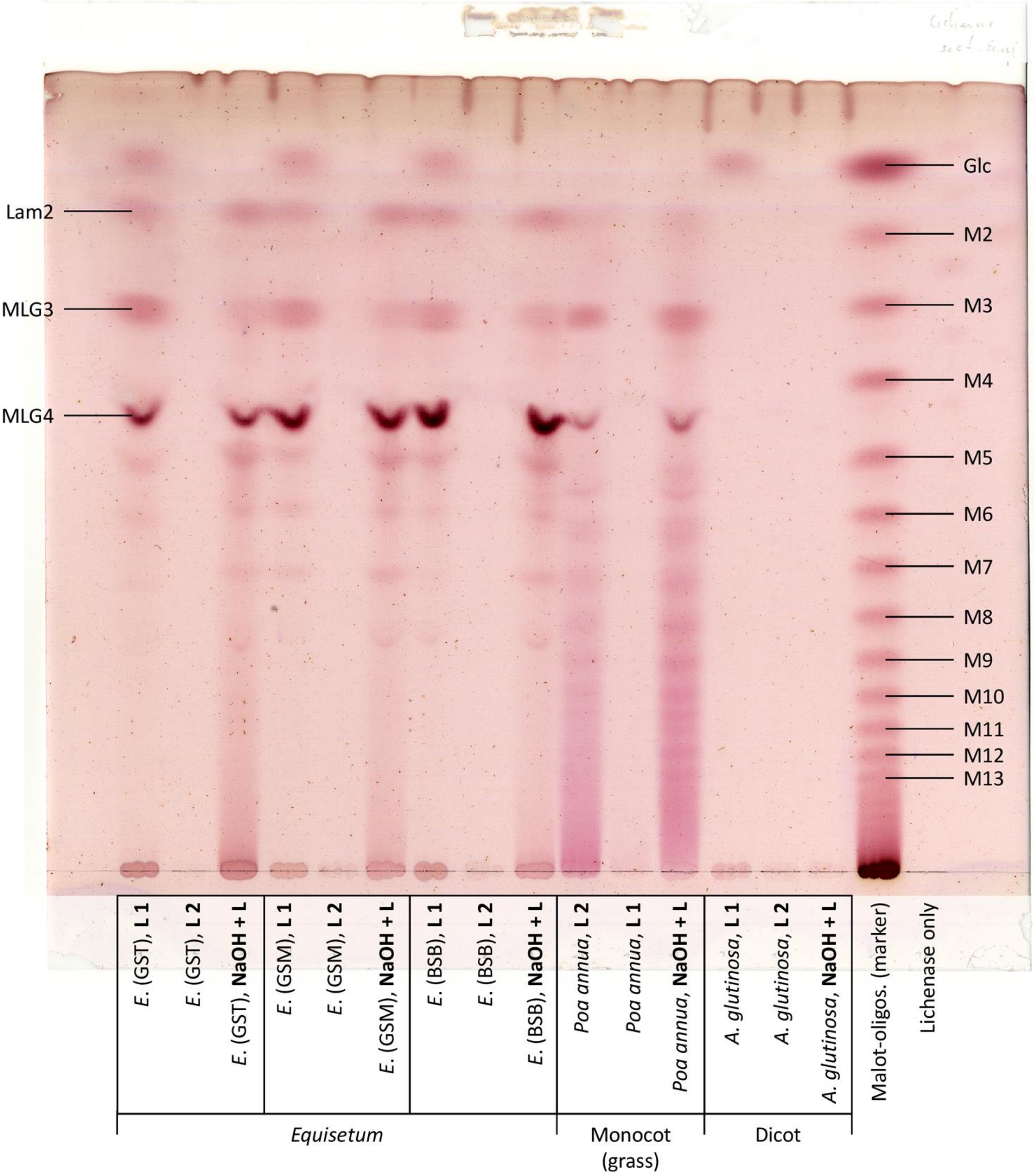

Supplemental Figure 1

Thin layer chromatography of lichenase digests of *Equisetum fluviatile*, *Poa annua* and *Alnus glutinosa* stems.

Sections were digested twice with lichenase (L1, L2) and digestion products released (soluble in 75% EtOH) were separated by thin-layer chromatography (butan-1-ol/acetic acid/water, 2:1:1) and stained with thymol/H_2SO_4. From a subsample of sections, hemicelluloses were extracted with NaOH, then neutralised and digested with lichenase (NaOH + L) and released products separated by thin-layer chromatography. GST, green shoot top; GSM, green shoot middle; BSB, black shoot base. Marker mixture contained malto-oligosaccharides. Glc, glucose; Lam2, laminaribiose (3-*O*-glucosyl-glucose); M2–M12, malto-oligosaccharides with degree of polymerisation 2–12. MLG3, 3-*O*-cellobiosyl-glucose; MLG4, 3-*O*-cellotriosyl-glucose.

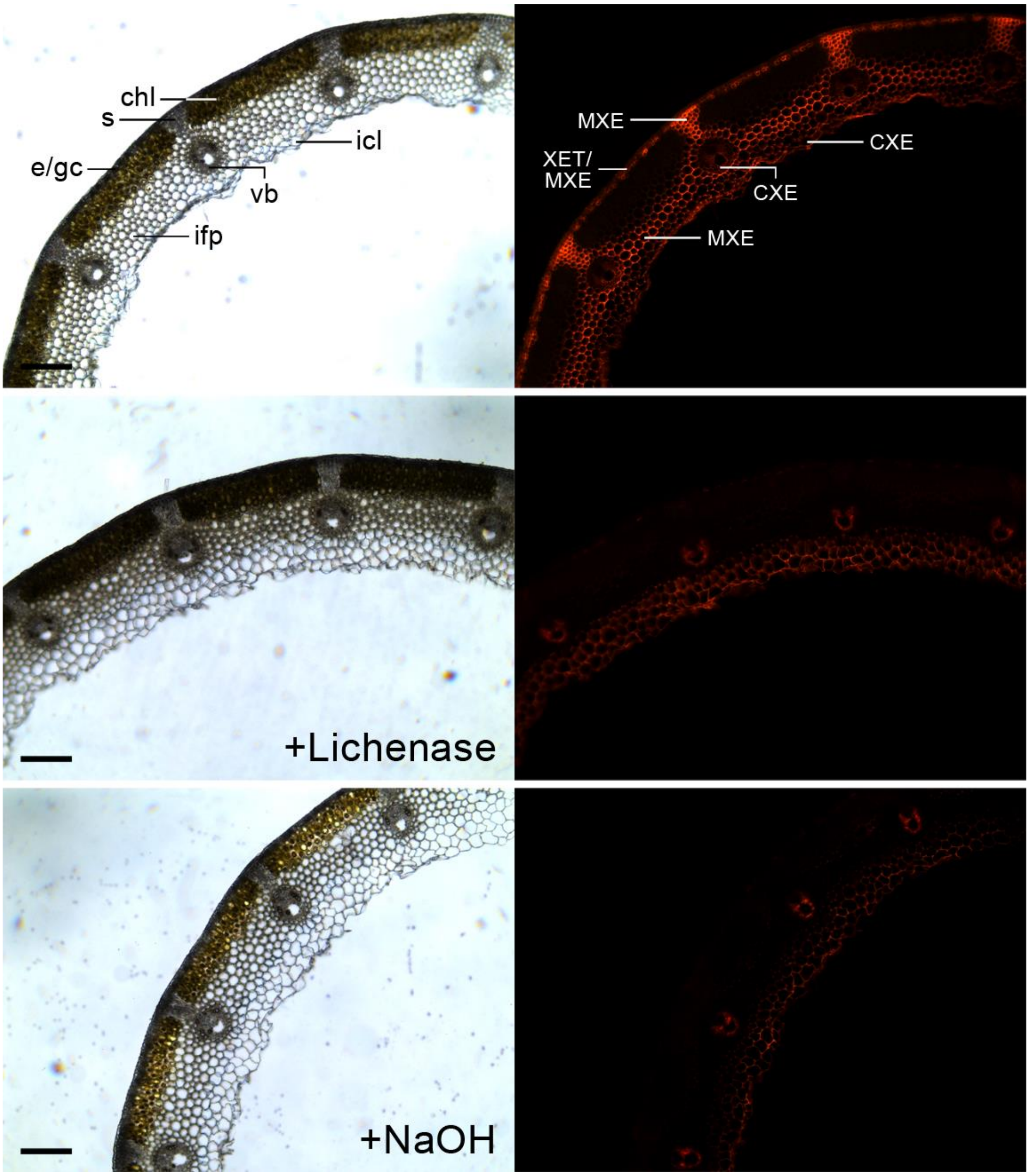

Supplemental Figure 2

Co-localisation of XET, MXE and CXE action and their endogenous donor substrates in young *Equisetum fluviatile* (GSM) internodes.

Bright field and corresponding fluorescence images showing XXXGol-sulforhodamine incorporated into cell walls of cross sections. After 4 h incubation with XXXGol-sulforhodamine, the sections were either simply washed (top; showing XET, MXE and CXE action), or washed and then digested with lichenase (middle; showing XET and CXE action), or washed and then treated with 6 M NaOH (bottom; showing only CXE action). Abbreviations: chl, chlorenchyma; e, epidermis; gc, guard cells; icl, inner cortex layer; ifp, interfascicular parenchyma; s, sclerenchyma; vb, vascular bundle. Scale-bar 250 µm.

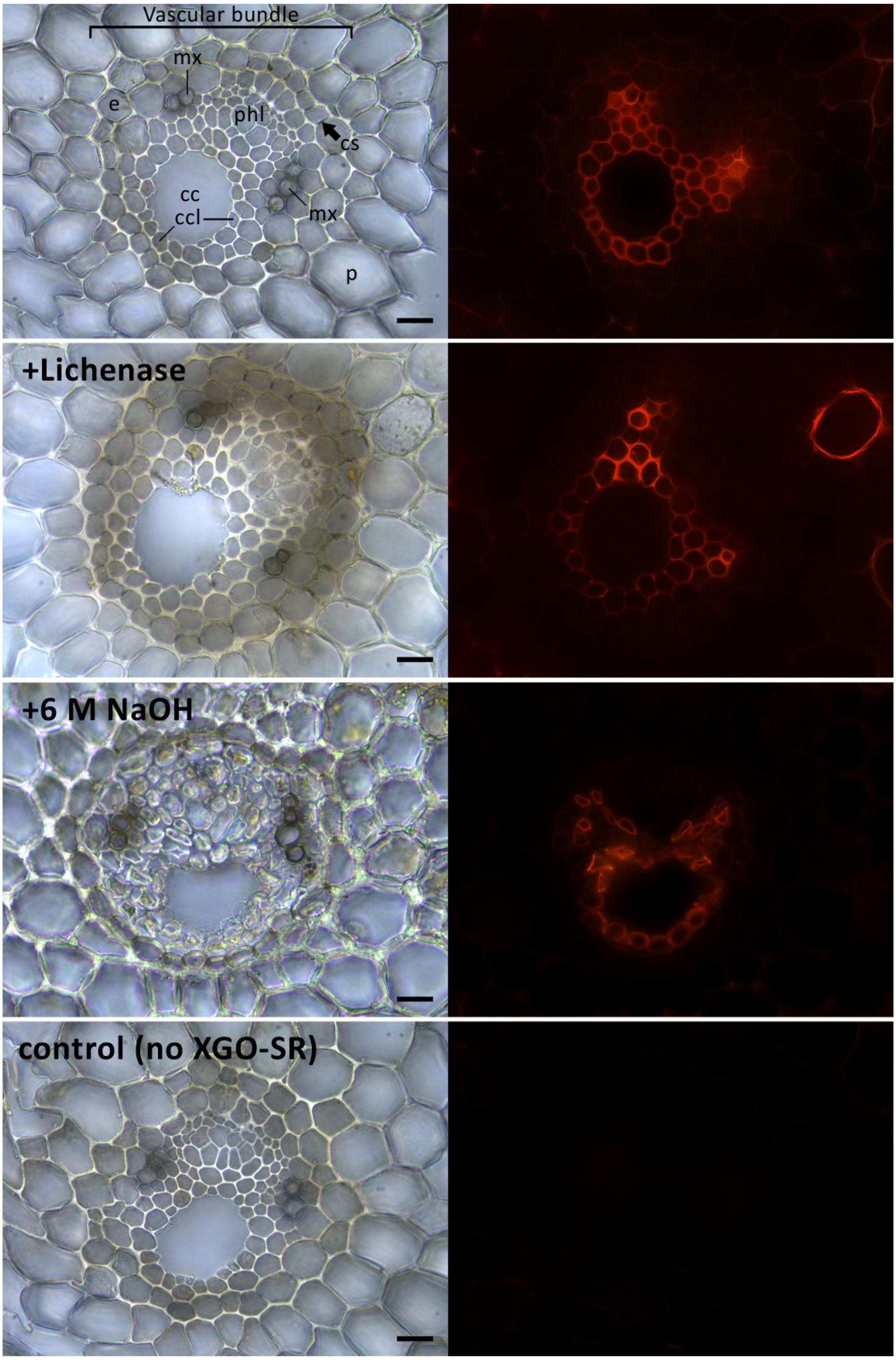

Supplemental Figure 3

Co-localisation of XET, MXE and CXE action and their endogenous donor substrates in vascular bundles in *Equisetum fluviatile* internodes (shoot base; BSB).

Methodology as in Supplemental Figure 6. Abbreviations: cc, carinal canal; ccl, carinal canal lining; cs, Casparian strip; e, endodermis; mx, metaxylem; p, parenchyma; phl, phloem. Scale-bar 25 µm.

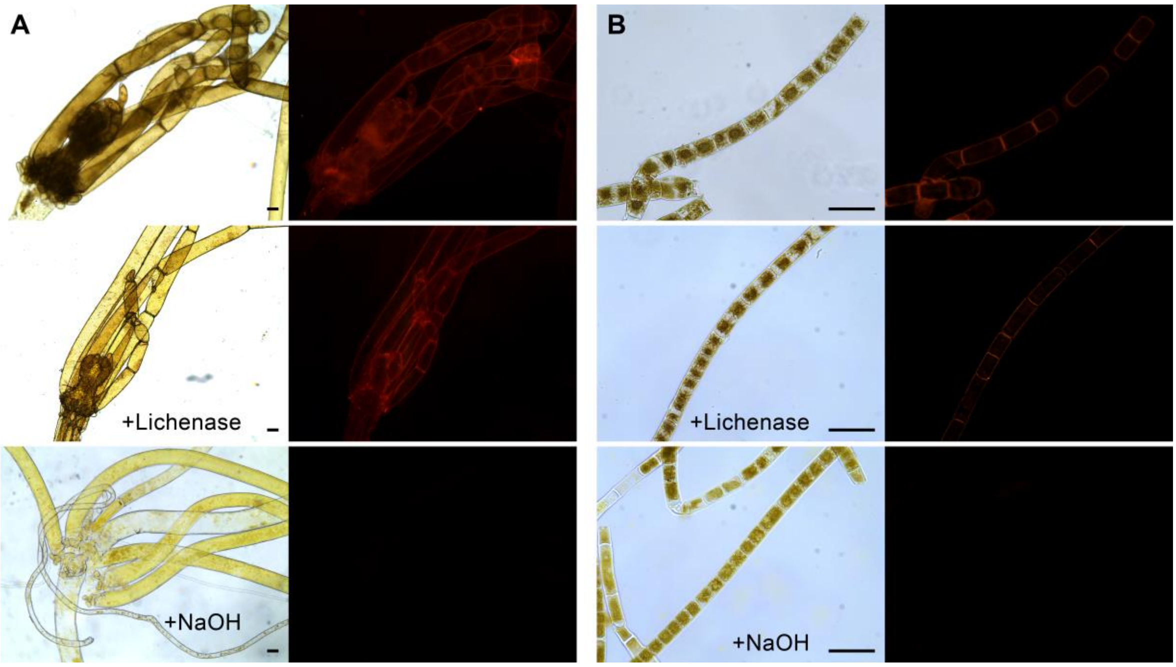

Supplemental Figure 4

Co-localisation of transglucanase action and the endogenous donor substrate(s) in charophytic green algae.

(A) *Chara vulgaris* thalli, (B) *Zygnema circumcarinatum* filaments. Methodology as in Supplemental Figure 6. Scale-bar 100 µm.

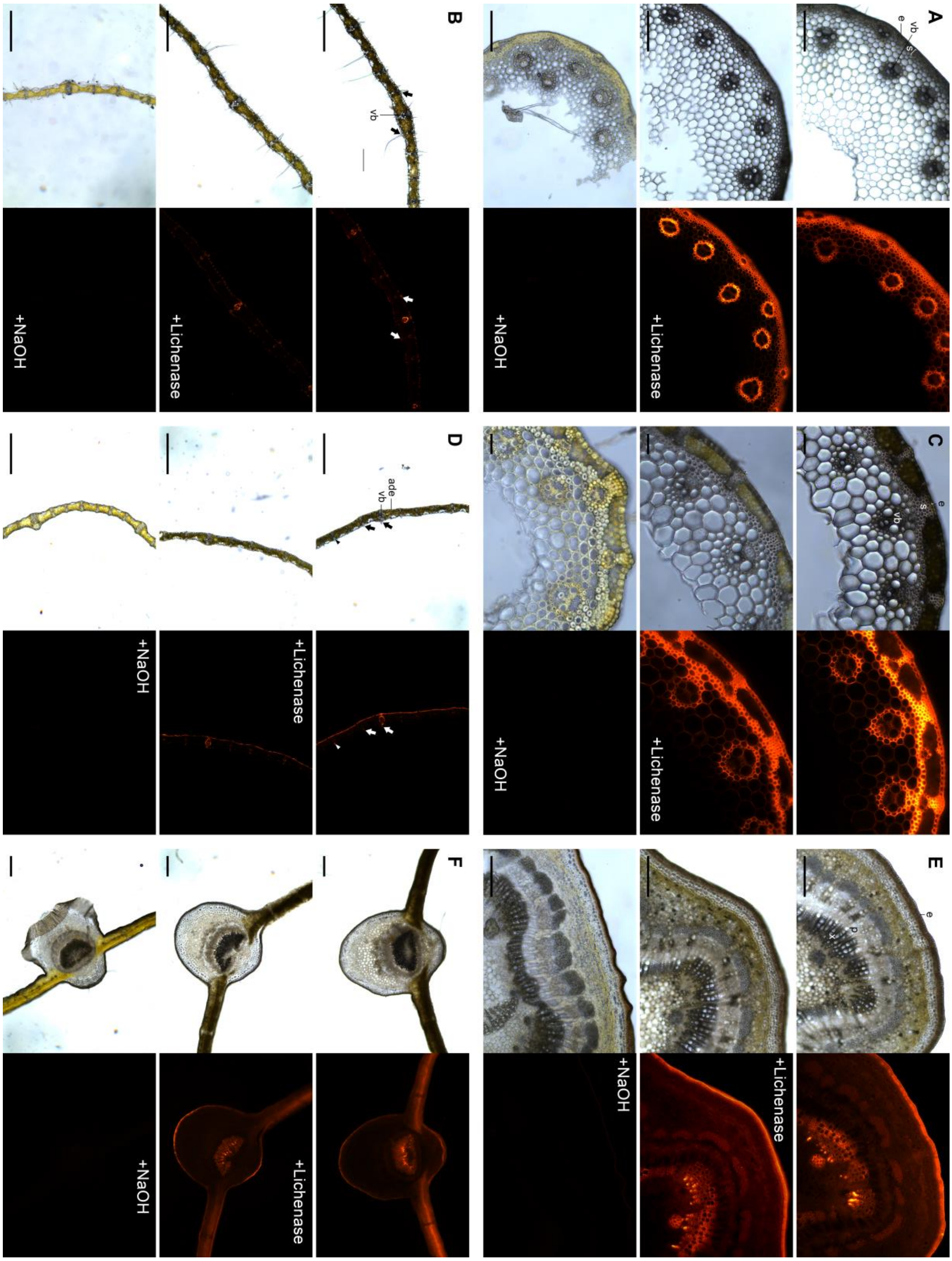

Supplemental Figure 5

Co-localisation of transglucanase action and the endogenous donor substrate(s) in leaves and stems of angiosperms.

Methodology as in Supplemental Figure 6. (A, B) Yorkshire fog grass, *Holcus lanatus*, (C, D) annual meadow grass, *Poa annua*, (E, F) alder tree, *Alnus glutinosa*. (A, C, E) Stems; (B, D, F) leaves, trichome base (arrows), sclerenchyma (arrowheads). Abbreviations: ade, adaxial epidermis; e, epidermis; s, sterome; vb, vascular bundle. Scale-bar 250 µm.

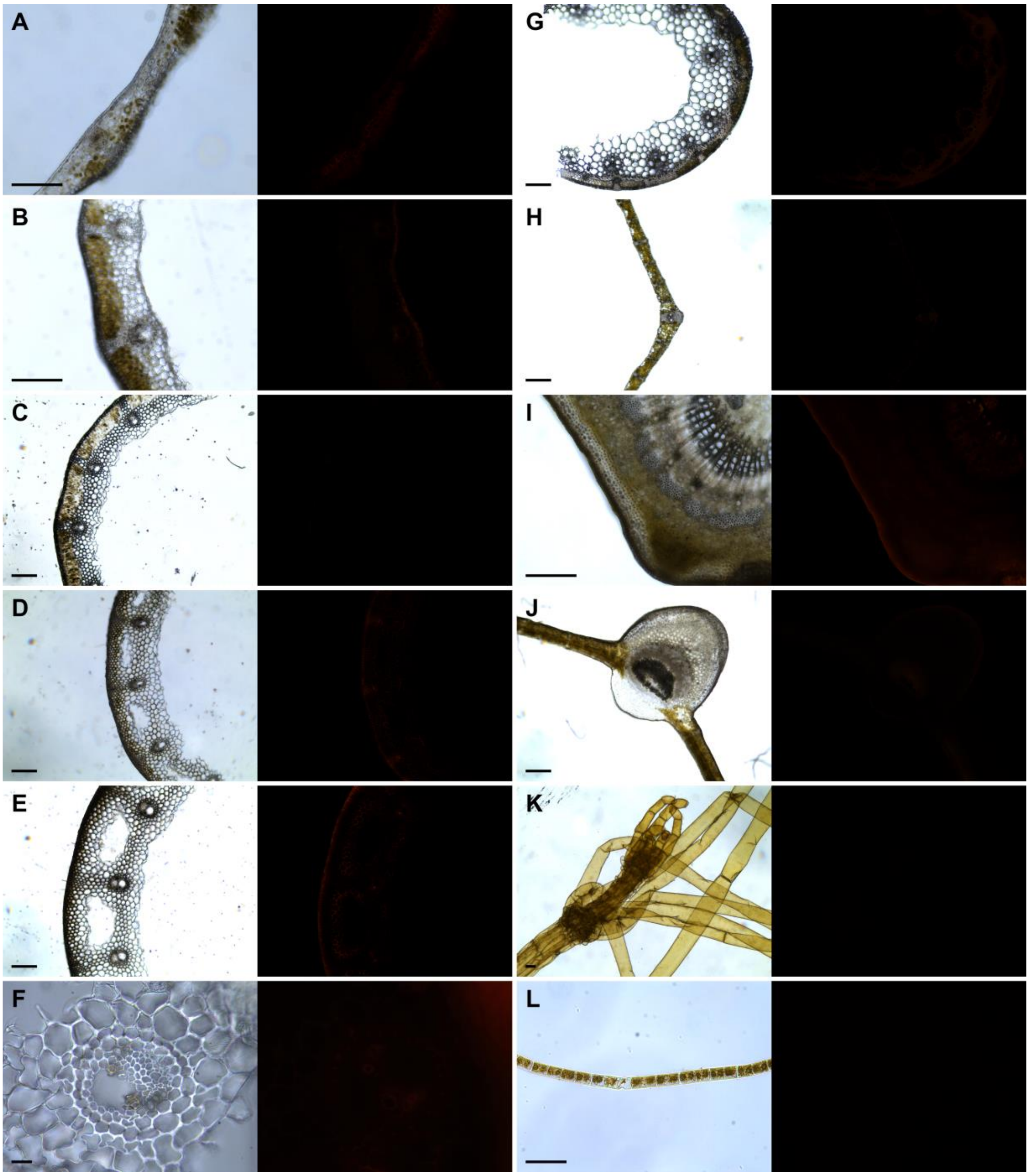

Supplemental Figure 6

Xyloglucan endoglucanase treatment solubilises essentially all products of endogenous transglucanase action on endogenous donor substrates in *Equisetum*, *Poa*, *Alnus* and charophyte green algae.

Cross sections or thalli were fed with XXXGol-sulforhodamine, followed by treatment with 0.5% (w/v) xyloglucan endoglucanase for 12 h, which removed fluorescent products by cleaving polysaccharide–XGO bonds. A contaminating enzyme in the xyloglucan endoglucanase probably partially hydrolysed MLG (see Supplemental Figure 7). (A) *Equisetum fluviatile* leaf, (B) young *Equisetum* internode (GST), (C) middle-aged *Equisetum* internode (GSM), (D) old *Equisetum* internode (shoot base; BSB), (E) old *Equisetum* internode (submerged; BSS), (F) vascular bundle in old *Equisetum* internode, (G) *Poa annua* stem, (H) *Poa* leaf, (I) *Alnus glutinosa* twig, (J) *Alnus* leaf, (K) *Chara vulgaris*, (L) *Zygnema circumcarinatum*. Scale-bar 250 µm (A–E, G–J), 25 µm (F, K, L).

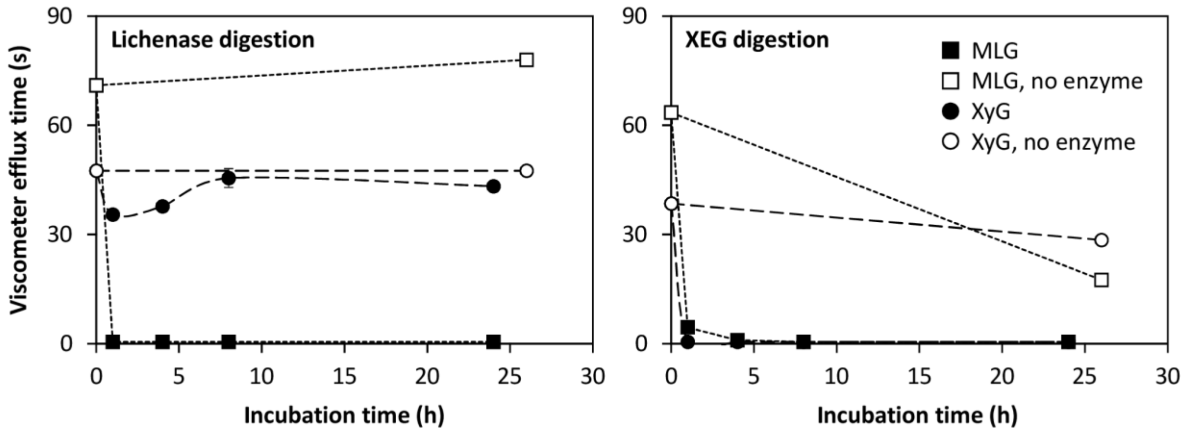

Supplemental Figure 7

Hydrolysis of MLG and xyloglucan by lichenase and xyloglucan endoglucanase, tested by a viscometric assay.

Buffered reaction mixtures contained 1% (w/v) polysaccharide (high-viscosity barley MLG or tamarind xyloglucan), 0.5 (w/v) chlorobutanol and either 10 units/ml lichenase in 100 mM citrate (pH 6.5) or 0.01% xyloglucan endoglucanase in pyridine/acetic acid/water, 1:1:98 (pH 4.7). Control assays lacked enzyme. Mixtures were sucked into a vertical 200-µl glass pipette and efflux time of 100 µl liquid was recorded. Samples were incubated at 25.4–25.8°C for up to 26 h (n=2±SD).

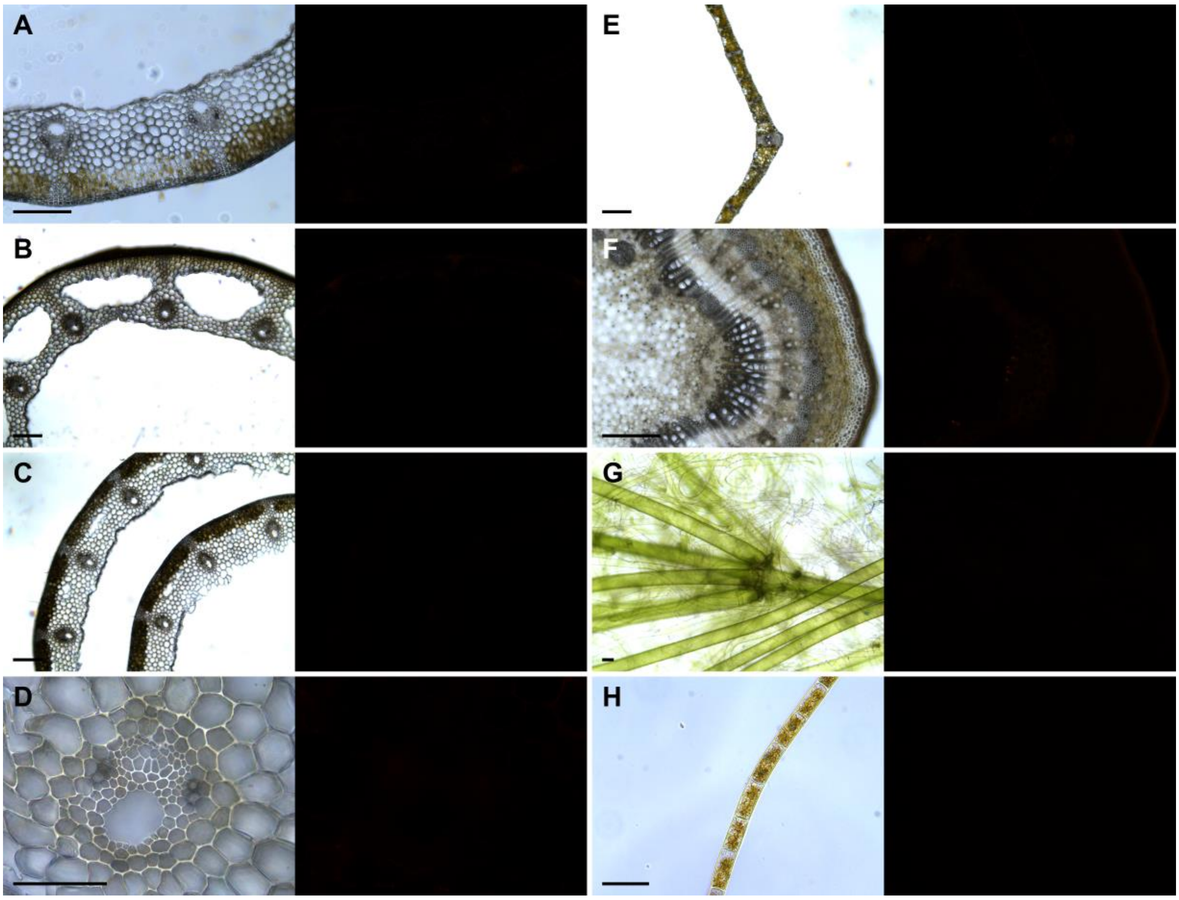

Supplemental Figure 8

Representative controls for co-localisation studies of transglucanase action and endogenous donor substrates (*in situ*).

Cross sections and thalli lack strong auto-fluorescence under the light settings applied for visualisation of XXXGol-sulforhodamine incorporation (excitation BP 546/12, emission BP 600/40). (A, B) Boiled cross sections (5 min; denaturing enzymes prior XXXGol-sulforhodamine feeding). (C-H) Non-boiled sections and thalli, where XXXGol-sulforhodamine was omitted from the reaction mixture. (A) Middle-aged *Equisetum* internode; GSM. (B) Old *Equisetum* internode (submerged; BSS). (C) Old *Equisetum* internode (shoot base; BSB). (D) Vascular bundle of old *Equisetum* internode. (E) *Poa annua* leaf, (F) *Alnus glutinosa* twig, (G) *Chara vulgaris*, (H) *Zygnema circumcarinatum*. Scale-bars 250 µm (A–C, E, F), 100 µm (D, G, H).

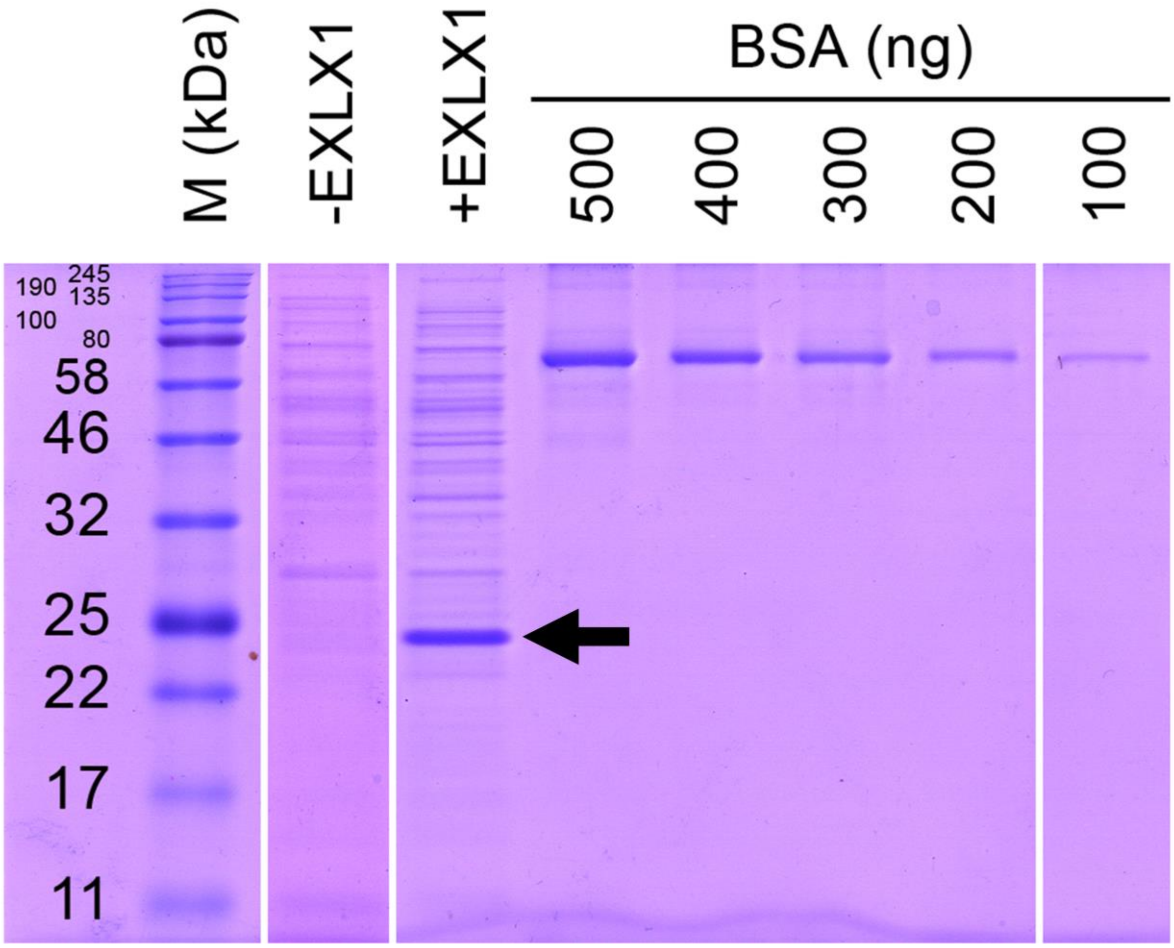

Supplemental Figure 9
SDS–PAGE gel.
Bacterial expansin (EXLX1) band (~23 kDa; arrow) in *E. coli* protein extracts and a BSA concentration gradient are shown.

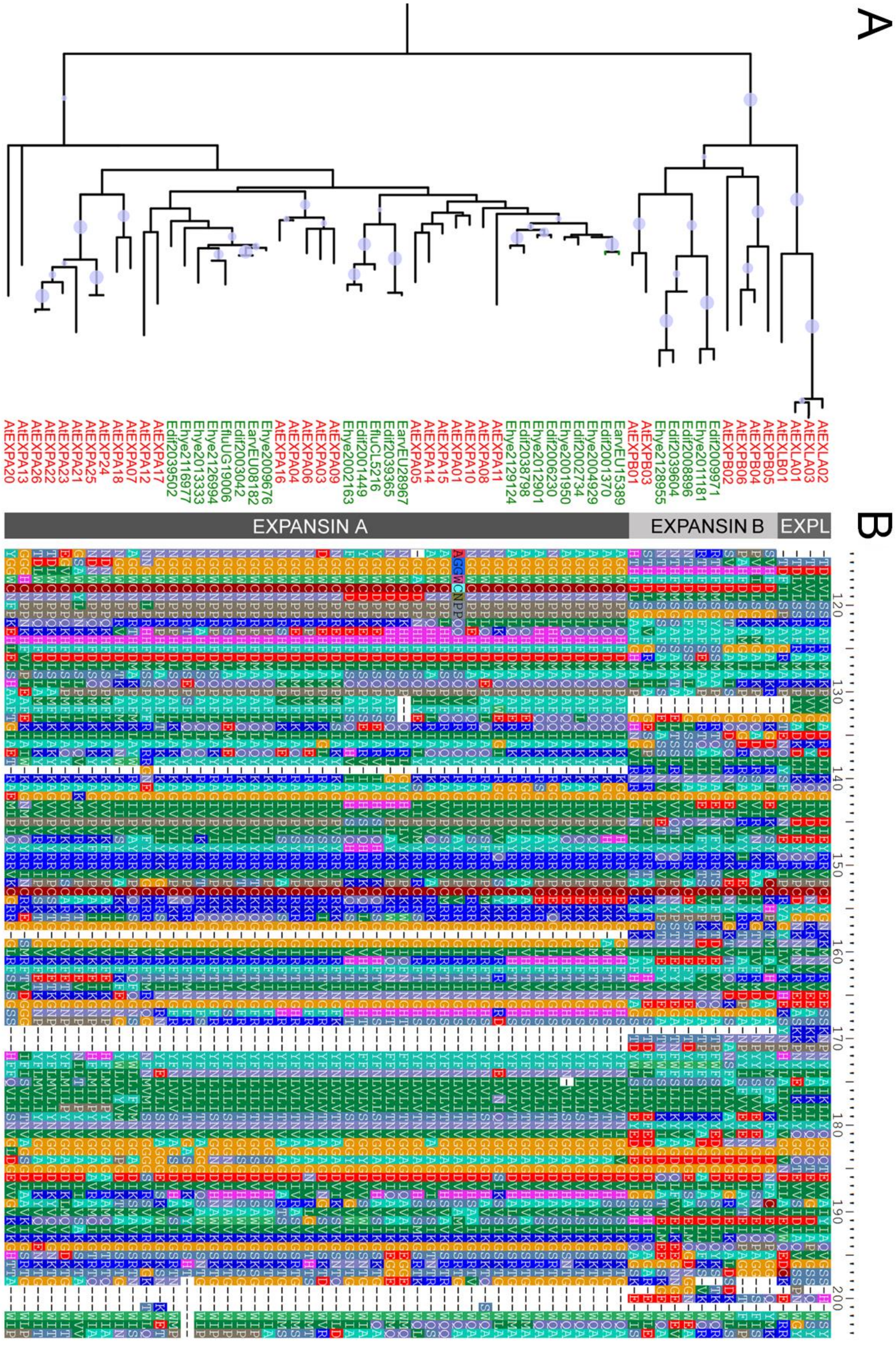
A
B
EXPANSIN A
EXPANSIN B
EXPL
AtEXPA20
AtEXPA13
AtEXPA26
AtEXPA22
AtEXPA23
AtEXPA21
AtEXPA25
AtEXPA24
AtEXPA18
AtEXPA07
AtEXPA12
AtEXPA17
Edif2039502
Ehye2116977
Ehye2013333
Ehye2126994
EfluUG19006
Edif2003042
EarvEU08182
Ehye2009676
AtEXPA16
AtEXPA04
AtEXPA06
AtEXPA03
AtEXPA09
Ehye2002163
Edif2001449
EfluCL5216
Edif2039365
EarvEU28967
AtEXPA05
AtEXPA14
AtEXPA15
AtEXPA01
AtEXPA10
AtEXPA08
AtEXPA11
Ehye2129124
Edif2038798
Ehye2012901
Edif2006230
Ehye2001950
Edif2002734
Ehye2004929
Edif2001370
EarvEU15389
AtEXPB01
AtEXPB03
Ehye2128955
Edif2039604
Edif2008896
Ehye2011181
Edif2009971
AtEXPB02
AtEXPB06
AtEXPB04
AtEXPB05
AtEXLB01
AtEXLA01
AtEXLA03
AtEXLA02
120
130
140
150
160
170
180
190
200

Supplemental Figure 10

Equisetum shoots express *EXPANSIN A* and *EXPANSIN B* genes

(A) Maximum Likelihood tree of EXPANSIN and EXPANSIN-LIKE (EXPL) proteins from *Arabidopsis thaliana* (red) and *Equisetum* species (green). *Equisetum* sequences were obtained from transcriptomes for developing shoots of *E. diffusum* (prefix Edif; www.onekp.com) sterile leaves and branches *E. hyemale* (Ehye; www.onekp.com), shoot apices of *E. arvense* (Earv; Frank *et al.*, 2015) or mature shoot tissue of *E. fluviatile* (Eflu; Simmons *et al.*, 2015). Because few of the *Equisetum* cDNAs appeared full-length, amino-acid sequences were aligned with MUSCLE and 90 positions (corresponding to residues 114-196 of *At*EXPA01, the product of At1g69530) that were represented in the majority of sequences used to estimate relationships. An LG model of substitution (Le *et al.*, 2008) with gamma-distributed rates among sites was used and implemented in Mega 10.0.5 (Kumar *et al.*, 2018). Nodes recovered in at least half of 250 bootstrap support are shown with circles with sizes proportional to the level of support. (B) Amino-acid sequence alignment used to estimate the protein tree in (a).

Supporting references:

Le, S.Q., Gascuel, O. (2008). An improved general amino acid replacement matrix. Mol. Biol. Evol. **25**:1307-1320.

Kumar, S., Stecher, G., Li, M., Knyaz, C., Tamura, K. (2018). MEGA X: molecular evolutionary genetics analysis across computing platforms. Mol. Biol. Evol. **35**:1547-1549.

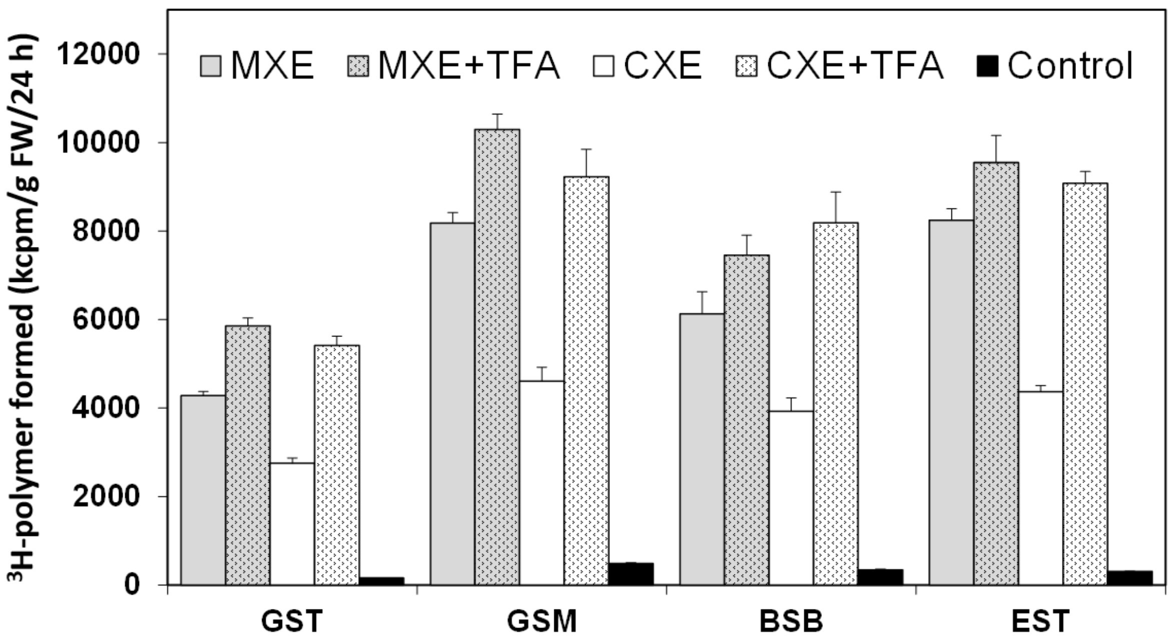

Supplemental Figure 11
Effect of acid hydrolysis on the counting efficiency of ^{3}H-labelled products of *Equisetum* MXE and CXE activities.
Protein extracts from various stem parts of August *Equisetum fluviatile* were assayed for MXE and CXE activity; the radioactive products (MLG–[^{3}H]XXXGol and cellulose–[^{3}H]XXXGol respectively) were further examined for ^{3}H detection efficiency. The donor substrate for CXE was alkali-pretreated paper (i.e. cellulose II, antiparallel arrangement). The control was equivalent to an MXE assay but with no donor substrate. Very low activities in control groups demonstrate that enzyme extracts contained only traces of co-extracted fern xyloglucan and MLG, which could also serve as donor substrates for XET or MXE activities, respectively, forming ^{3}H-labelled hemicelluloses that would remain attached to the paper after removal of free [^{3}H]XXXGol by water-washing. After the assay, the MXE products or control samples were dried onto paper and washed in running tap-water, whereas the CXE products were washed in alkali then water. CXE product remains stable after thorough washing with 6 M NaOH, whereas XET and MXE products would have been solubilised. MXE and CXE papers were dried and assayed for radioactivity in water-immiscible scintillation fluid (Gold Star). Next the paper-bound products, freed of scintillant, were heated in 2 M TFA at 120°C for 1 h and dried. The hydrolysis products were redissolved in water and re-assayed for ^{3}H by scintillation-counting at 33% counting efficiency in water-miscible scintillation fluid (OptiPhase HiSafe 3). The data demonstrate that hot TFA disintegration of the test papers used in MXE and CXE *in-vitro* assays can enhance the counting efficiency of ^{3}H-labelled products. This indicates that *in-vitro* CXE products (and therefore the CXE activity) can be underestimated if assayed without the TFA treatment. Hot TFA-treatment of CXE test papers resulted in a ~100% increase in counts per minute (cpm), whereas MXE test papers increased by only 10–20%. In conclusion, the extracted enzyme exhibited approximately equal activities of MXE and CXE when the counting efficiency was optimised.
